# Somatic evolution following cancer treatment in normal tissue

**DOI:** 10.1038/s41586-025-09792-4

**Published:** 2025-12-10

**Authors:** Oriol Pich, Sophia Ward, Andrew Rowan, Cristina Naceur-Lombardelli, Oliver Shutkever, Carlos Martinez-Ruiz, Siân Harries, Sonya Hessey, Babu Naidu, James D. Brenton, John Le Quesne, Anne Thomas, Cathy Richards, Matthew G. Krebs, Samra Turajlic, Sanjay Jogai, Simone Zaccaria, David Moore, Crispin T. Hiley, PEACE Consortium, Oriol Pich, Sophia Ward, Andrew Rowan, Cristina Naceur-Lombardelli, Oliver Shutkever, Carlos Martinez-Ruiz, Siân Harries, Sonya Hessey, Babu Naidu, James D. Brenton, Cathy Richards, Matthew G. Krebs, Samra Turajlic, Sanjay Jogai, Simone Zaccaria, David Moore, Crispin T. Hiley, John Le Quesne, Kai-Keen Shiu, John Bridgewater, Daniel Hochhauser, Martin Forster, Siow Ming Lee, Tanya Ahmad, Dionysis Papadatos-Pastos, Sam Janes, Peter Van Loo, Katey Enfield, Ariana Huebner, Sergio Quezada, Stephan Beck, Tariq Enver, David R. Pearce, Mary Falzon, Ron Sinclair, Zoe Rhodes, Teresa Marafioti, Miriam Mitchison, Mark Linch, Sebastian Brandner, Selvaraju Veeriah, Heather Shaw, Gerhardt Attard, Faye Gishen, Maise Al-Bakir, Nnenna Kanu, Francisco Gimeno-Valiente, Emilia L. Lim, James Reading, Benny Chain, Adrienne Flanagan, Emma Colliver, Mihaela Angelova, James Black, Olivia Lucas, William Hill, Wing Kin Liu, Alexander Frankell, Roberto Salgado, Kristiana Grigoriadis, Takahiro Karasaki, Abigail Bunkum, Sarah Benafif, Vittorio Barbè, Supreet Kaur Bola, Osvaldas Vainauskas, Anna Wingate, Daniel Wetterskog, A. M. Mahedi Hasan, Stefano Lise, Gianmarco Leone, Anuradha Jayaram, Constantine Alifrangis, Ursula McGovern, Kerstin Thol, Samuel Gamble, Seng Kuong Ung, Piotr Pawlik, Roberto Vendramin, Jayant Rane, Angela Dwornik, Kerry Bowles, Jeanette Kittel, Kerstin Haase, Rija Zaidi, Athanasia Vargiamiou, Lucrezia Patruno, Christopher Aled Chamberlain, Welles Robinson, Iain McNeish, Nataly Ojeda Mosquera, Jiali Liu, Felix O’Farrell, Chenelle Marcel, James Larkin, Lisa Pickering, Andrew Furness, Kate Young, Will Drake, Kim Edmonds, Nikki Hunter, Mary Mangwende, Lauren Grostate, Lavinia Spain, Scott Shepherd, Haixi Yan, Benjamin Shum, Zayd Tippu, Brian Hanley, Charlotte Spencer, Max Emmerich, Camille Gerard, Eleanor Carlyle, Steve Hazell, Hardeep Mudhar, Christina Messiou, Arash Latifoltojar, Annika Fendler, Fiona Byrne, Husayn Pallikonda, Irene Lobon, Alexander Coulton, Anne-Laure Cattin, Daqi Deng, Hugang Feng, Nadia Yousaf, Sanjay Popat, Charlotte Milner-Watts, Emma Nye, Aida Murra, Justine Korteweg, Lauren Terry, Jennifer Biano, Kema Peat, Emma Turay, Peter Hill, Marija Miletic, Anadil Javaid, Jennifer Thomas, Bakir Kudic, Orla McGowan, Dharmista Ramesh, Oznur Saka, Sinem Arslan, Laura Marandino, Reina Ammar, Gurneet Kapur, Dilruba Kabir, David McMahon, Alexius John, Foteini Kalofonou, Debra Josephs, Sheeba Irshad, James Spicer, Anna Green, Ruby Stewart, Natasha Wright, Ruxandra Mitu, Deborah Enting, Sarah Rudman, Sharmistha Ghosh, Eleni Karapanagiotou, Elias Pintus, Andrew Tutt, Nicola Thompson, Rebecca Fitzgerald, Merche Jimenez-Linan, Elena Provenzano, Anna Paterson, Kieren Allinson, Grant D. Stewart, Ultan McDermott, Tim Maughan, Olaf Ansorge, Peter Campbell, Patricia Roxburgh, Sioban Fraser, Kevin Blyth, Fiona Blackhall, Yvonne Summers, Pedro Oliveira, Caroline Dive, Fabio Gomes, Mat Carter, Dean Fennell, Jacqui Shaw, Claire Wilson, Charlotte Poile, Kudazyi H. Kutywayo, Maurice R. Dungey, Jens Claus Hahne, Shobhit Baijal, Gerald Langman, Charlotte Ferris, Hollie Bancroft, Amy Kerr, Gary Middleton, Joanne Webb, Salma Kadiri, Bernard Olisemeke, Rodelaine Wilson, Aya Osman, Ian Tomlinson, Judith Cave, Luke Nolan, Samantha Holden, Tania Fernandes, Dave Chuter, Mairead McKenzie, Allan Hackshaw, Aoife Walker, Hayley Bridger, Rachel Leslie, Shivani Patel, Charles Swanton, Mariam Jamal-Hanjani, Nicholas McGranahan, Charles Swanton, Mariam Jamal-Hanjani, Nicholas McGranahan

**Affiliations:** 1 Cancer Evolution and Genome Instability Laboratory, The Francis Crick Institute, London, UK; 2 Cancer Research UK Lung Cancer Centre of Excellence, University College London Cancer Institute, London, UK; 3 Genomics Science Technology Platform, The Francis Crick Institute, London, UK; 4 Cancer Metastasis Laboratory, University College London Cancer Institute, London, UK; 5 University College London Hospitals, London, UK; 6 Cancer Genome Evolution Research Group, University College London Cancer Institute, London, UK; 7 Computational Cancer Genomics Research Group, University College London Cancer Institute, London, UK; 8 Birmingham Acute Care Research Group, Institute of Inflammation and Ageing, University of Birmingham, Birmingham, UK; 9 Cancer Research UK Cambridge Institute, University of Cambridge, Li Ka Shing Centre, Cambridge, UK; 10 Cancer Research UK Major Centre–Cambridge, University of Cambridge, Cambridge, UK; 11 Cambridge University Hospital NHS Foundation Trust and National Institute for Health Research Cambridge Biomedical Research Centre, Addenbrooke’s Hospital, Cambridge, UK; 12 Cancer Research UK Scotland Institute, Glasgow, UK; 13 School of Cancer Sciences, University of Glasgow, Glasgow, UK; 14 NHS Greater Glasgow and Clyde Pathology Department, Queen Elizabeth University Hospital, Glasgow, UK; 15 Leicester Cancer Research Centre, College of Life Sciences, University of Leicester, Leicester, UK; 16 University Hospitals of Leicester NHS Trust, Leicester, UK; 17 Division of Cancer Sciences, The University of Manchester, Manchester, UK; 18 The Christie NHS Foundation Trust, Manchester, UK; 19 National Institute for Health and Care Research (NIHR) Manchester Biomedical Research Centre (BRC), Manchester, UK; 20 The Royal Marsden Hospital, London, UK; 21 The Institute of Cancer Research, London, UK; 22 Cancer Dynamics Laboratory, The Francis Crick Institute, London, UK; 23 Cancer Dynamics Laboratory, The CRUK Manchester Institute, Manchester, UK; 24 NIHR Biomedical Research Centre, School of Clinical and Experimental Sciences, Faculty of Medicine, University of Southampton, Southampton, UK; 25 University Hospital Southampton NHS Trust, Southampton, UK; 26 Department of Cellular Pathology, University College London Hospitals, London, UK; 27 University College London Cancer Institute, London, UK; 28 UCL Respiratory, Division of Medicine, University College London Hospitals, London, UK; 29 Department of Genetics, The University of Texas MD Anderson Cancer Center, Houston, TX USA; 30 Department of Genomic Medicine, The University of Texas MD Anderson Cancer Center, Houston, TX USA; 31 Department of Pathology and Laboratory Medicine, University of British Columbia, Vancouver, British Columbia Canada; 32 Basic and Translational Research, BC Cancer Research Institute, Vancouver, British Columbia Canada; 33 Immune Regulation and Tumour Immunotherapy Group, Cancer Immunology Unit, Research Department of Haematology, University College London Cancer Institute, London, UK; 34 Medical Genomics, University College London Cancer Institute, London, UK; 35 University College London Queen Square Institute of Neurology, London, UK; 36 Mount Vernon Cancer Centre, Northwood, UK; 37 UCL Medical School, University College London, London, UK; 38 The Whittington Hospital NHS Trust, London, UK; 39 Department of Biochemistry and Molecular Biology and Edwin SH Leong Centre for Healthy Aging, University of British Columbia, Vancouver, British Columbia Canada; 40 The University of British Columbia, Vancouver, British Columbia Canada; 41 Pre-Cancer Immunology Laboratory, Cancer Research UK Lung Cancer Centre of Excellence, University College London Cancer Institute, London, UK; 42 Department of Pathology, ZAS Hospitals, Antwerp, Belgium; 43 Division of Research, Peter MacCallum Cancer Centre, Melbourne, Victoria Australia; 44 Department of Thoracic Surgery, Respiratory Center, Toranomon Hospital, Tokyo, Japan; 45 Human Biology Facility–Organoids Service, The Francis Crick Institute, London, UK; 46 Imperial College London, London, UK; 47 St Bartholomew’s Hospital, Barts Health NHS Trust, London, UK; 48 Queen Mary University of London, London, UK; 49 The Francis Crick Institute, London, UK; 50 Sheffield Teaching Hospitals NHS Foundation Trust, Sheffield, UK; 51 Experimental Histopathology, The Francis Crick Institute, London, UK; 52 Imperial College London NHS Foundation Trust, London, UK; 53 Guy’s and St Thomas’ NHS Foundation Trust, London, UK; 54 King’s College London, London, UK; 55 Addenbrooke’s Hospital, Cambridge University Hospitals, Cambridge, UK; 56 Early Cancer Institute, Department of Oncology, University of Cambridge, Cambridge, UK; 57 Department of Surgery, University of Cambridge, Cambridge, UK; 58 Wellcome Sanger Institute, Hinxton, UK; 59 MRC Oxford Institute for Radiation Oncology, University of Oxford, Oxford, UK; 60 Beatson West of Scotland Cancer Centre, Glasgow, UK; 61 Pathology department, Queen Elizabeth University Hospital, Glasgow, UK; 62 Division of Cancer Sciences, The University of Manchester and The Christie NHS Foundation Trust, Manchester, UK; 63 Cancer Research UK Lung Cancer Centre of Excellence, University of Manchester, Manchester, UK; 64 CRUK Manchester Centre, Manchester, UK; 65 Cancer Research UK National Biomarker Centre, Cancer Research UK Manchester Institute, University of Manchester, Manchester, UK; 66 University of Leicester, Leicester, UK; 67 Leicester Medical School, University of Leicester, Leicester, UK; 68 National Institute for Health Research Biomedical Research Centre and Cancer Research UK Experimental Cancer Medicine Centre, University of Leicester, Leicester, UK; 69 University Hospital Birmingham NHS Foundation Trust, Birmingham, UK; 70 Institute of Immunology and Immunotherapy, University of Birmingham, Birmingham, UK; 71 Department of Oncology, University Hospital Southampton NHS Foundation Trust, Southampton, UK; 72 Independent Cancer Patients’ Voice, London, UK; 73 Cancer Research UK and UCL Cancer Trials Centre, London, UK

**Keywords:** Cancer genomics, Cancer therapy

## Abstract

The extent to which exogenous sources, including cancer treatment, contribute to somatic evolution in normal tissue remains unclear. Here we used high-depth duplex sequencing^1^ (more than 30,000× coverage) to analyse 168 cancer-free samples representing 16 organs from 22 patients with metastatic cancer enroled in the PEACE research autopsy study. In every sample, we identified somatic mutations (range 305–2,854 mutations) at low variant allele frequencies (median 0.0000323). We extracted 16 distinct single-base substitution mutational signatures, reflecting processes that have moulded the genomes of normal cells. We identified alcohol-induced mutation acquisition in liver, smoking-induced mutagenesis in lung and cardiac tissue, and multiple treatment-induced processes, which correlated with therapy type and duration. Exogenous sources, including treatment, underpinned, on average, more than 40% of mutations in liver but less than 10% of mutations in brain samples. Finally, we observed tissue-specific selection, with positive selection in tissues such as lung (*PTEN* and* PIK3CA*), liver (*NF2L2*) and spleen (*BRAF* and* NOTCH2*), and limited selection in others, such as brain and cardiac tissue. More than 25% of driver mutations in normal tissue exposed to systemic anti-cancer therapy, including in *TP53*, could be attributed to treatment. Immunotherapy, although not associated with increased mutagenesis, was linked to driver mutations in *PPM1D* and *TP53*, illustrating how non-mutagenic treatment can sculpt somatic evolution. Our study reveals the rich tapestry of mutational processes and driver mutations in normal tissue, and the profound effect of lifetime exposures, including cancer treatment, on somatic evolution.

## Abstract (web summary)

High-depth sequencing of non-cancerous tissue from patients with metastatic cancer reveals single-base mutational signatures of alcohol, smoking and cancer treatments, and reveals how exogenous factors, including cancer therapies, affect somatic cell evolution.

## Main

High-depth mutational profiling studies have revealed that histologically normal tissue can carry a patchwork of driver alterations. This work has also uncovered a positive relationship between age and the size and number of mutant clones across a range of cancer-free tissues^2–4^, including skin^5,6^, bladder^7^, colon^8^, liver^9^, oesophagus^10,11^ and brain^12^. However, most of these studies have only considered mutagenesis in the context of healthy ageing and naturally occurring mutational exposure (for example, UV light).

Recent work has revealed that cancer treatment can act as a source of mutagens, leading to an increased acquisition of both passenger and driver alterations in tumours^13^. This raises important questions regarding the effect of treatment not only in the context of cancer, but also in normal tissue. There is a need to understand the extent to which the mutagenic insults of treatment, combined with different exogenous exposures, affect different normal tissues, and, moreover, whether non-mutagenic treatments, including immunotherapy, can act as selection pressures, shaping somatic evolution. Here we harness a unique resource of normal tissue and blood samples from patients treated with different cancer therapies, coupled with highly sensitive duplex sequencing^1^, to explore and evaluate somatic evolution and the effect of treatment in normal tissue.

## Somatic mutations in normal tissue

**Fig. 1:**
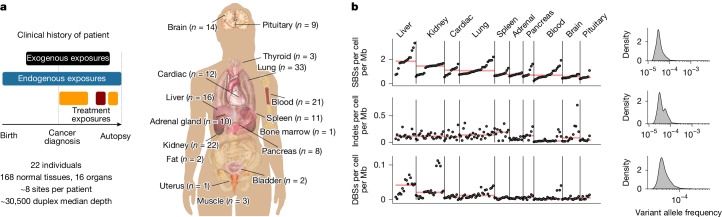
Cohort to explore effect of treatment on normal tissue. **a**, Overview of the cohort and sample collection. Images from 10.15347/wjm/2014.008 (Mikael Häggström, CC0 1.0). **b**, Estimated number of mutations per Mb per cell for each tissue type and VAF density distribution.

Posthumous Evaluation of Advanced Cancer Environment (PEACE; ClinicalTrials.gov ID NCT03004755; 13/LO/0972) is a national, multicentre, pan-cancer research autopsy programme. To investigate the effect of cancer treatment and other exogenous agents across normal tissues both within and between individuals, we obtained 156 2 mm × 2 mm fresh frozen biopsies from 16 different types of cancer-free normal tissues (including pituitary, spleen and thyroid that have not previously been subject to deep sequencing), in addition to 22 blood samples and 5 metastatic tumour samples, from 22 patients with metastatic disease enroled in PEACE (Fig. 1a). For two patients, in addition to cancer-free tissue from autopsy, normal tissue samples collected prior to cancer treatment were available through the TRACERx study (ClinicalTrials.gov ID NCT01888601). Thus, for 22 patients, 158 samples were obtained at autopsy (153 samples from normal tissue and 5 cancer samples), 20 blood samples were obtained after primary treatment and 5 samples (2 blood and 3 normal tissue samples) were obtained prior to cancer therapy at the point of surgical resection of a primary tumour.

**Extended Data Fig. 1:**
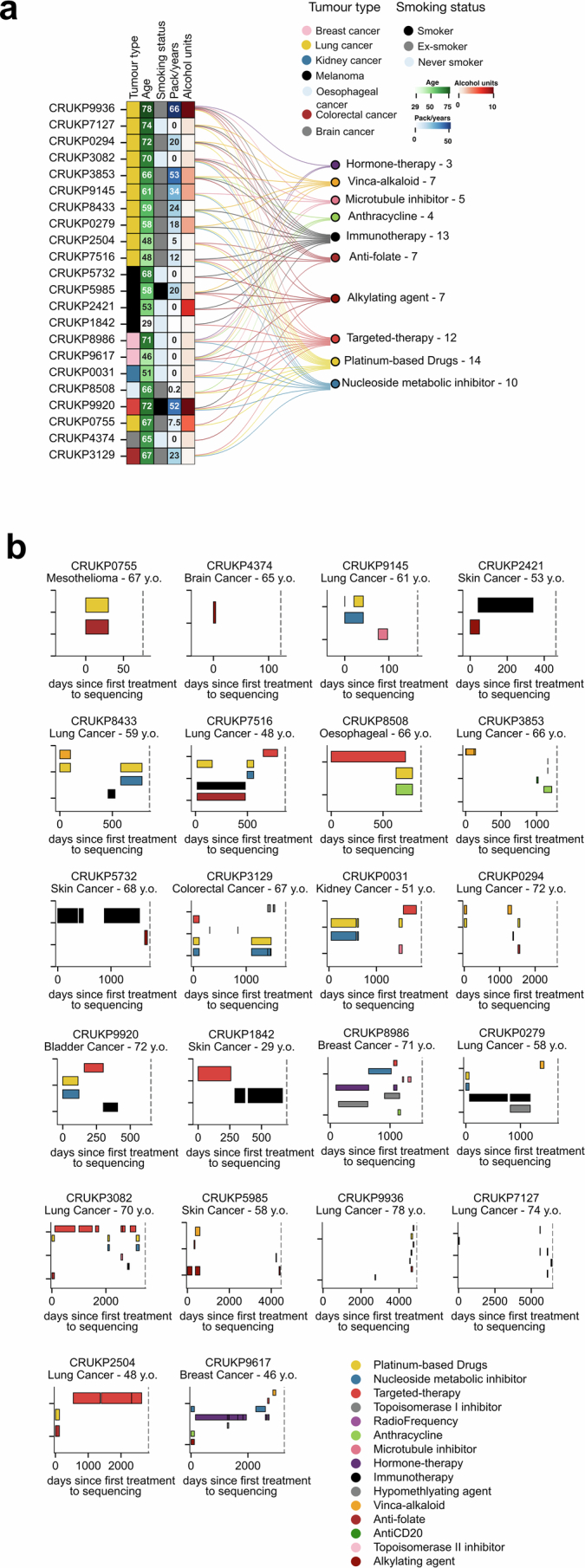
**A**. Clinical details and treatment. Alcohol units reflect self-reported consumption per week. **B**. The treatment history of each patient within our cohort is depicted, ranging from the first day of treatment exposure until the patient died. Treatment duration is represented as rectangles, colored by the class of cancer therapy.

The median age of the cohort was 65-years (range 29–78 years) and individuals were diagnosed with a range of cancers (Supplementary Table 1). Critically, we had access to detailed patient records, including treatment history and potential exogenous exposures (such as tobacco and alcohol) that may influence the acquisition of somatic alterations (Extended Data Fig. 1 and Supplementary Table 1). As a whole, the cohort received a range of therapies, including chemotherapy, immunotherapy and targeted therapies (Extended Data Fig. 1).

**Extended Data Fig. 2:**
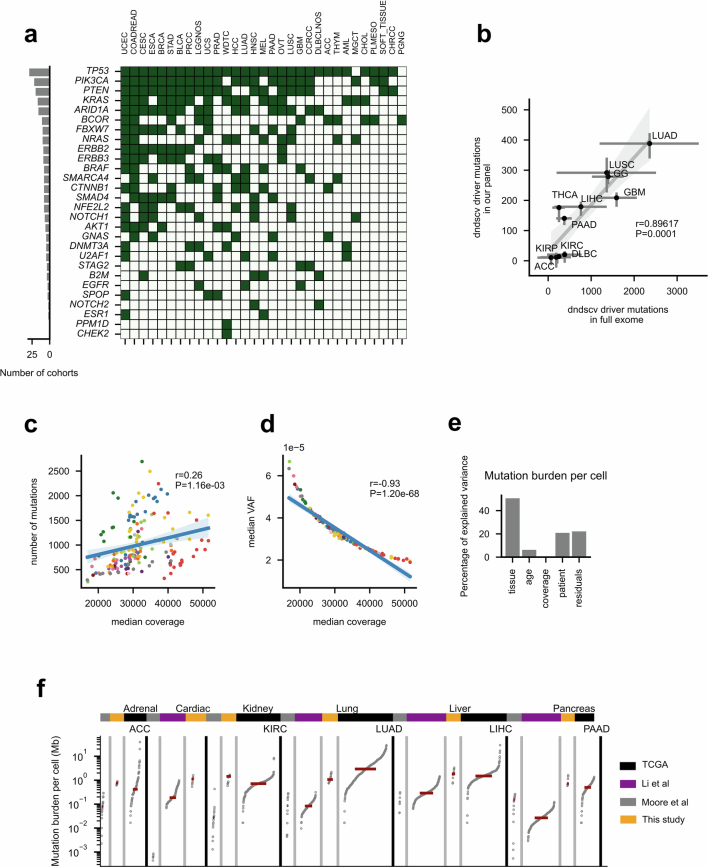
**A**. Rows represent the genes selected in our panel, and columns represent tumour cohorts within the IntoGen database^14^. Dark green shows that the gene is under positive selection in the respective cohort. **B**. Correlation (n = 9) between the global dNdScv ratios in TCGA tumours limiting the observed mutations to our designed panel (y axis) and the full exome (x axis). Vertical and horizontal lines represent the respective confidence intervals. The matched normal-tumours are: ‘’LGG, GBM”:Brain, “PAAD”:Pancreas, “KIRC, KIRP”:Kidney, “LICH”:Liver, “ACC”:Adrenal, ‘THCA’:Thyroid, “LUAD,LUSC”:’Lung’, ‘DLBC’:Blood. **C**. Correlation between the number of mutations and the median duplex coverage. Each dot represents a sample, colored by each tissue type (legend in Fig. 1). **D**. Correlation between the median VAF of the observed mutations in each sample and the respective median coverage. Each dot represents a sample, colored by each tissue type (legend in Fig. 1). **E**. Barplot representing how much tissue, patient, age, coverage and the residuals explain, in percentage, the median mutation burden per cell, using a linear model. **F**. Distribution of mutation burden per Mb per cell across different tumour types from TCGA (Adrenocortical carcinoma (ACC), Kidney renal clear cell carcinoma (KIRC), Lung adenocarcinoma (LUAD), Liver Hepatocelular carcinoma (LIHC), Pancreatic adenocarcinoma (PAAD, black rectangle) compared to the corresponding normal tissue across different projects (this study, Moore et al.^4^, Li et al.^3^). PAAD is compared to normal pancreas, LIHC is compared to liver, LUAD is compared to Lung, KIRC to kidney, ACC to adrenal. Cardiac tissue is also represented without a match tumour type. Red horizontal line represents the median mutation burden.

To interrogate positive selection, acquisition of driver alterations and the mutational processes that act on normal tissue, we designed a 82.5-kb duplex sequencing panel that covered 30 known cancer genes (including *TP53*, *EGFR*, *KRAS* and *BRAF*)^14^ (Extended Data Fig. 2a,b and Supplementary Table 2), as well as highly mutagenic genomic regions, selected to provide an unbiased sampling of sequence contexts (GC content, genic or non-genic, and coding or non-coding). To achieve high sensitivity and specificity for somatic variant detection we utilized duplex sequencing, an error-correction method that uses unique molecular identifiers coupled with information from reads derived from both original strands of a DNA molecule, thus eliminating artefactual alterations and enabling mutation quantifications with a low error rate^1,15^ (see Supplementary Note for comparison with NanoSeq^16^).

In total, we performed duplex sequencing on 156 normal tissue samples, 22 blood samples and 5 tumour samples (median 30,504× coverage per sample; interquartile range (IQR) 27,684–36,450) (Fig. 1a,b). For 171 samples, the 82.5-kb custom panel was used for sequencing, whereas for 12 samples, a smaller mutagenesis panel was used (Methods). These 12 samples were only used for mutational signature analysis. Ten samples were excluded owing to potential cancer cell contamination or quality control (Methods). No samples were taken from the same organ (or bilateral organ in the case of lungs and kidney) as a site of metastasis.

We identified 166,732 somatic single-base substitutions (SBSs), 16,108 insertion–deletion mutations (indels) and 2,399 double-base substitutions (DBSs) in the cohort (Fig. 1b). The majority of mutations were identified at low variant allele frequencies (VAFs; median VAF = 0.0000323, IQR 0.000027−0.000042; Fig. 1b), indicating their presence in a limited number of cells. We observed a significant positive correlation between the number of mutations and median sequencing coverage (*r* = 0.26, *P* = 1.16 × 10^−3^, Extended Data Fig. 2c) and a significant negative correlation between median VAF and coverage (*r* = −0.93, *P* = 1.20 × 10^−68^, Extended Data Fig. 2d). Thus, to obtain an estimate of the number of mutations per megabase (Mb) per cell, independent of sequencing depth, we integrated VAFs with the number of mutations and the length of the sequencing panel (Methods). Variance in mutations per Mb per cell could be substantially explained by tissue type (50%, *P* = 4.7 × 10^−24^), patient (20%, *P* = 3.26 × 10^−10^) and age (6%, *P* = 3.26 × 10^−10^) (Extended Data Fig. 2e).

Compared with other tissues, liver samples were characterized by a higher burden of mutations per Mb per cell (1.83, IQR 1.71–2.14), equivalent to clonal populations of untreated hepatocellular carcinoma cells (Extended Data Fig. 2f). By contrast, brain and pituitary tissue were characterized by a lower mutation burden of 0.58 (IQR 0.47–0.64) and 0.50 (IQR 0.47–0.54) per Mb per cell, respectively. Notably, our sequencing approach does not permit cell-type deconvolution, and thus heterogeneity between and within samples may also reflect different cell-type composition.

## Mutational signatures in normal samples

Given the variation in mutations per Mb per cell across tissues and individuals (Extended Data Fig. 2e), we next investigated the mutational processes that sculpt the genomes of normal cells and how these might relate to exposures. We performed de novo mutational signature analysis on all samples using a Bayesian hierarchical–Dirichlet process^17^ (Methods), separately considering mutations resulting from SBSs, IDs and DBSs.

**Fig. 2:**
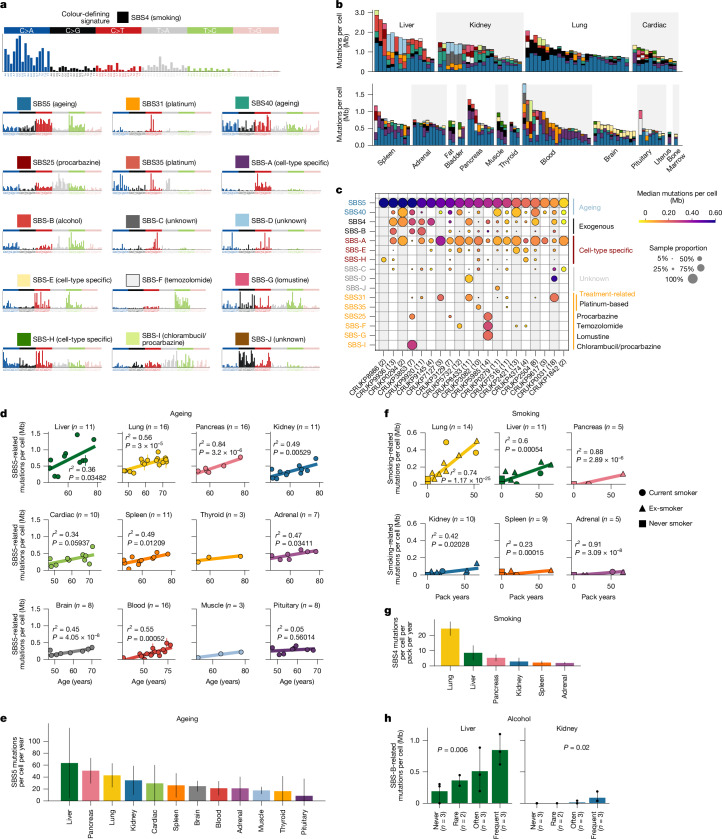
Mutational processes that are active in treated normal tissue. **a**, Mutational signatures identified with SBSs. **b**, Prevalence of each mutational signature in samples across tissues. Colours correspond to signatures in **a**. **c**, Median mutation rate of each signature in each patient. Some patients are characterized by highly specific treatment-related signatures. **d**, Correlation between SBS5 mutational signature and patient age for each tissue type, assessed using robust linear models. When multiple samples from the same patient are present, the median is used and the vertical line connects the minimum and maximum values. **e**, Estimated number of SBS5 mutations acquired per genome per cell per year for each tissue type. Lines represent the 95% confidence intervals. **f**, Correlation assessed using robust linear model between pack years and number of estimated smoking-related SBS4 mutations per Mb per cell for tissues exhibiting evidence of smoking-mediated mutagenesis. When multiple samples fom the same patient are present, the median is used and the vertical line connects the minimum and maximum values. **g**, Estimated number of SBS4 mutations per genome per cell per pack year. Lines represent the 95% confidence intervals. **h**, Relationship between alcohol consumption and number of SBS-B mutations in liver and kidney samples. Significance was assessed using Jonckheere–Terpstra test. Dots represent samples. Lines connect minimum and maximum values.

**Extended Data Fig. 3:**
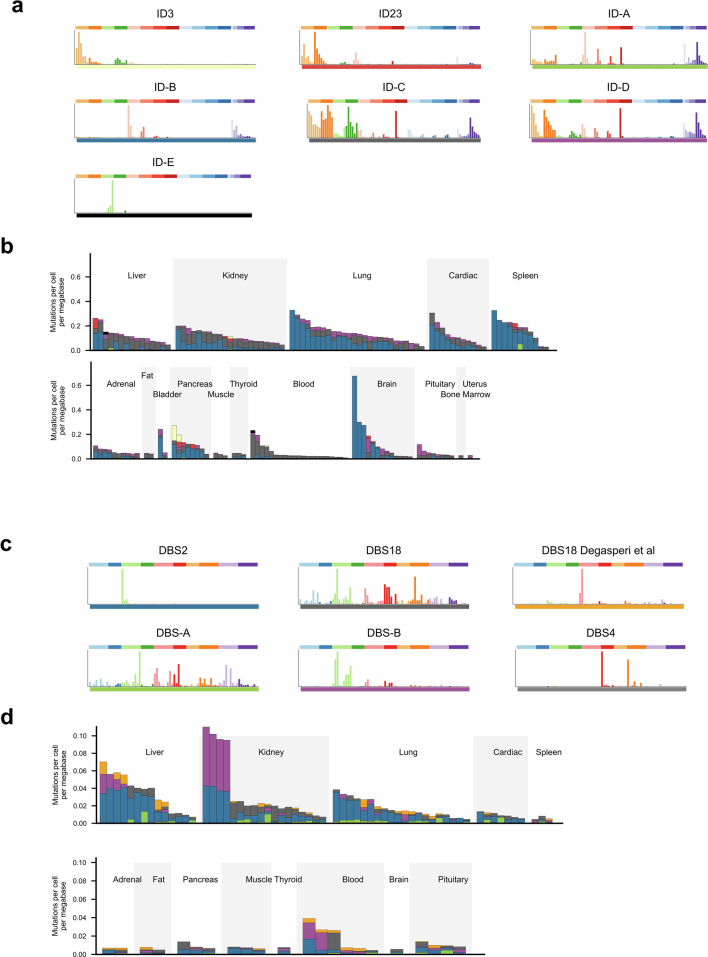
**A**. Mutational signatures identified through analysis with indels **B**. Prevalence of ID signatures. **C**. Double base-substitutions, DBS, signatures. **D**. Prevalence of DBS signatures.

In total, we identified 16 distinct single-base substitution (SBS) mutational signatures (Fig. 2a). Six of these could be linked to previously described Catalogue of Somatic Mutations in Cancer (COSMIC)^18^ signatures, reflecting smoking (SBS4), age-related clock-like processes (SBS5 and SBS40), or treatment-induced mutagenesis (SBS31, SBS35 and SBS25). We also identified ten SBS signatures (denoted as SBS-A to SBS-J), which have not currently been described in COSMIC (Fig. 2a). Likewise, seven indel and six DBS signatures were identified (Extended Data Fig. 3), of which two indel (ID3 and ID23) and two DBS (DBS2 and DBS18) signatures have been described in COSMIC (Supplementary Table 4 provides an overview of all mutational signatures identified).

## Ageing and lifestyle mutational processes

**Extended Data Fig. 4:**
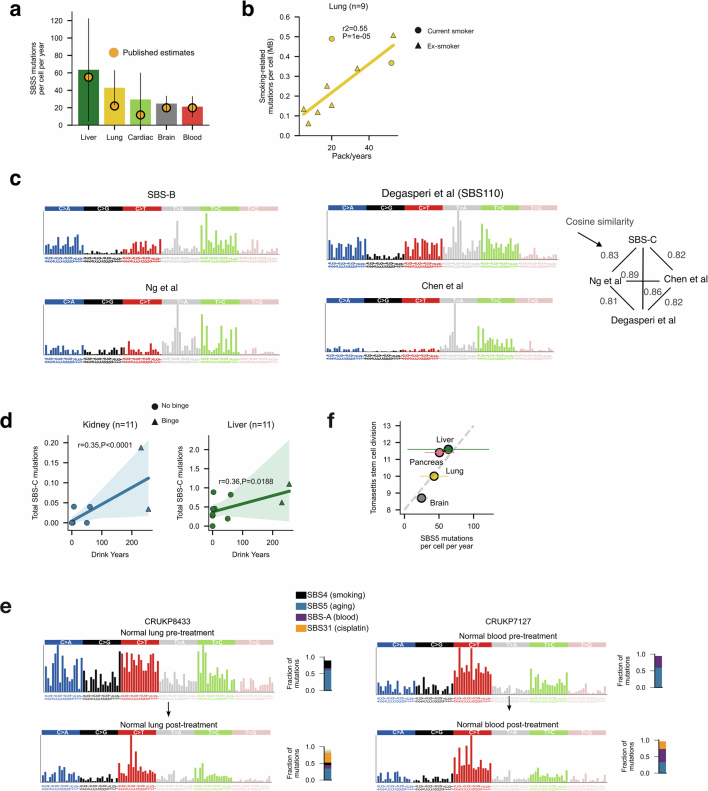
**A**. Comparison of SBS5 mutations per cell per year to estimates obtained in previously published work^16,57–59^. Bars represent confidence intervals for our cohort, **B**. Robust linear correlation between SBS4 mutations per cell per Mb and pack years after excluding samples with no exposure to SBS4 and never-smokers. **C**. Mutational signature profile of SBS-B, a liver-specific signature from Ng et al.^21^, a liver specific signature from Chen et al.^22^, and a signature found in Degasperi in Genomics England in liver cancers^23^. The cosine similarity across the four profiles is also depicted. **D**. Correlation between SBS-B mutations per cell per year/MB and drink-year estimates in kidney and liver samples. **E**. Paired samples before and following treatment with cisplatin. Both trinucleotide channel plots from all mutations and estimated mutational signatures (barplot) are depicted. **F**. Correlation between the estimated number of SBS5 mutations per cell per year and the stem cell divisions per year from Tomasetti^26^.

The most pervasive SBS mutational signature across the cohort was SBS5, which contributed to 40% of mutations per sample on average (IQR 26–52%) (Fig. 2b,c). Consistent with previous work^19^, we observed a significant relationship between patient age and SBS5 mutation burden (Fig. 2d). The relationship between SBS5 and age varied significantly depending on tissue (*P* = 1.252 × 10^−13^), consistent with varying rates of SBS5 mutation acquisition per cell per year across tissues (Fig. 2e). In lung, we estimated that around 43 SBS5 mutations were acquired per cell per year, compared to approximately 24 and 21 in blood and brain samples, respectively (Fig. 2e). Our results are broadly consistent with previous studies, with discordance potentially reflecting differences in the cell composition of the sequenced samples and/or alternative sequencing approaches (Extended Data Fig. 4a).

Certain mutational signatures were preferentially identified in specific tissues. SBS4, characterized by C>A transversions and linked to tobacco-mediated mutagenesis^20^, was identified in lung, liver and cardiac tissues (Fig. 2b). In all three tissues, we observed a significant relationship between pack years (reflecting the number of packets of cigarettes smoked per day multiplied by the number of years the individual has smoked) and SBS4 mutations (Fig. 2f and Extended Data Fig. 4b). These data suggest that for every pack year, the cells in the lung accumulate approximately 20 mutations, whereas cardiac and liver cells accumulate around 5 mutations (Fig. 2g). This relationship remained significant when restricted to ex-smokers (*R*^2^ = 0.55, *P* = 0.0005), suggesting that there is a reservoir of histologically normal cells with smoking-related mutations, despite smoking cessation.

Other predominantly tissue-specific signatures included SBS-B, a mutational process characterized predominantly by T>C transitions and a T>A peak at CpTpG sites. SBS-B was identified in 15 out of 16 liver samples, contributing up to 38% of mutations per liver sample in which it was detected (range 10–38%). This signature bears similarity to published signatures described in the context of chronic liver disease^21^ and hepatocellular carcinomas^22,23^ (Extended Data Fig. 4c). In liver tissue, the burden of SBS-B mutations per Mb was significantly associated with alcohol consumption (as measured by self-assessed alcohol units) (*P* = 0.006, Jonckheere–Terpstra test; Fig. 2h) and inferred drink years (Extended Data Fig. 4d and Methods).

We also identified tissue-specific signatures that have been described in the context of normal tissue. SBS-A was enriched in blood samples, and exhibited a similar trinucleotide context to a signature identified in cord blood, granulocytes and haematopoietic stem cells^16,24^ (cosine similarity 0.96). The presence of a small proportion of SBS-A mutations in most tissues may reflect blood infiltration across samples. SBS-E was similar (cosine similarity 0.93) to a signature previously identified in neurons^16^ and was enriched in samples from brain and pituitary tissue (Fig. 2b).

## Mutational footprints of therapies

Using the detailed clinical history of the cohort, we directly evaluated the relationship between treatment and mutational signatures. We identified multiple mutational signatures that could be linked to therapy.

**Fig. 3:**
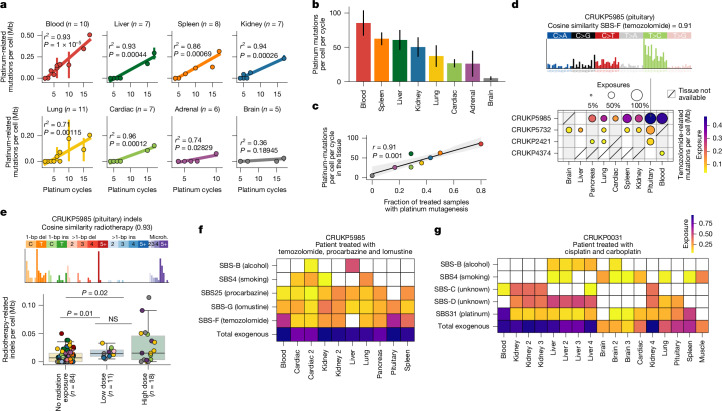
Treatment-related mutational signatures in normal tissues. **a**, Correlation between number of platinum cycles and estimated platinum-related mutations per cell (SBS31 and 35), assessed using robust linear models. When multiple samples from the same patient are present, the median is used and the vertical line connects the minimum and maximum values. **b**, Estimated number of platinum-related mutations per genome per cell per platinum treatment cycle in a given tissue. Lines represent the 95% confidence intervals. **c**, Pearson correlation between the number of platinum-related mutations induced per cycle in the tissue and the fraction of treated samples with platinum mutagenesis detected. **d**, Trinucleotide context mutational profile plot for representative pituitary sample treated with temozolomide (top) and heat map showing number of SBS-F related mutations in each tissue treated with temozolomide in four patients (bottom). Only tissues that were sequenced in at least two samples are represented in the heat map. **e**, Association between indel signature ID-E and radiation treatment. Significance assessed using Mann–Whitney *U* test. Del, deletions; ins, insertions; microh., microhomology mutations. NS, not significant. **f**, Fraction of mutations linked to distinct exogenous mutational processes across CRUKP5985 samples. **g**, Fraction of mutations linked to distinct exogenous mutational processes across CRUKP0031 samples.

We observed clear evidence for platinum-mediated mutagenesis in samples from patients treated with platinum chemotherapy (Extended Data Fig. 4e), with a statistically significant positive relationship between the number of platinum cycles and the burden of signatures SBS31 and SBS35 (Fig. 3a,b). Blood samples exhibited the highest rate of platinum mutagenesis (89 mutations per cell per platinum cycle), and the lowest was observed in brain and adrenal tissue (fewer than 27 mutations per cell per platinum cycle) (Fig. 3b). This may conceivably be explained by the fact that certain cells or tissues are more directly exposed to treatment, or that certain cell types have an increased propensity to repair mutagenic insults^25^ (Fig. 3c). This may also be influenced by the relative cell turnover of different tissues^26^ (Extended Data Fig. 4f).

**Extended Data Fig. 5:**
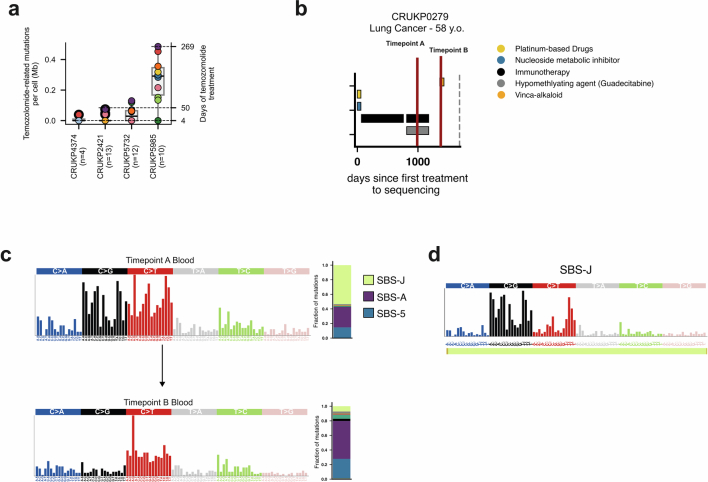
**A**. Relationship between temozolomide-related mutations per cell per Mb and the days of temozolomide treatment. Each dot represents a sample. **B**. Treatment history of CRUKP0279. Two time-points at which blood was sampled are highlighted in red. **C**. Top, the mutational spectra of single-base substitutions at time point A, with the signature exposures depicted on the right. Bottom, the mutational spectra of single-base substitutions at time point B, and the signature exposures depicted on the right. **D**. Mutational profile of SBS-J.

We also identified non-COSMIC mutational signatures associated with specific treatments. In 18 out of 39 samples from the 4 patients treated with temozolomide there was evidence of mutations associated with SBS-F, a signature similar to one described previously^27^, reflecting temozolomide-induced mutagenesis with intact mismatch repair (Fig. 3d). A linear mixed-effect model revealed a significant association between the number of temozolomide-related signature mutations and both tissue type and number of temozolomide treatment days (*P* = 0.03, *P* = 2.64 × 10^−5^, respectively; Extended Data Fig. 5a). Spleen, pituitary and cardiac tissue were associated with the greatest burden of temozolomide-related mutations (median 20%, range 5%–43%). Similarly, we identified the SBS-I signature in a patient treated with chlorambucil, suggesting that this alkylating agent also leaves a unique genomic scar^28^. ID-E, an indel signature similar to one described in the context of radiation^29^, was significantly enriched in samples exposed to radiotherapy, particularly in brain, pituitary and lung tissue, with on average 0.04 indels per cell per Mb detected per sample (Fig. 3e). SBS-J, a mutational signature characterized by C>G transversions, was identified at high prevalence exclusively in a blood sample from a patient undergoing treatment with the hypomethylating agent guadecitabine (Extended Data Fig. 5b–d). The high prevalence of this signature may reflect the mutational footprint of guadecitabine on cycling cells.

Analyses of individual patients illustrated how different treatments can profoundly sculpt the genomes of normal tissue throughout an individual’s life. For instance, in samples from patient CRUKP5985, a 57-year-old man who was treated for 2 cancers over a period of 13 years (initial glioblastoma multiforme diagnosis followed by malignant melanoma 6 years later), we identified a total of 8 distinct mutational signatures, of which 3—SBS-F, SBS25 and SBS-G—could be linked to treatment (Fig. 3f). The mutational footprints of temozolomide (SBS-F) were identified in all samples, contributing more than 35% of mutations in the blood and pituitary samples. SBS25, a signature linked to the chemotherapy drug procarbazine^30–32^, was identified in 9 out of 10 samples (median contribution, 13.74%). This patient had received 6 cycles of procarbazine, lomustine (also known as CCNU) and vincristine, consistent with SBS25 reflecting DNA damage from procarbazine treatment. We also uniquely identified SBS-G, a novel signature, which exhibited a preponderance of T>G, C>G and C>T mutations, in all samples from this individual, which may reflect a signature of DNA damage associated with lomustine treatment, an alkylating nitrosourea compound.

**Extended Data Fig. 6:**
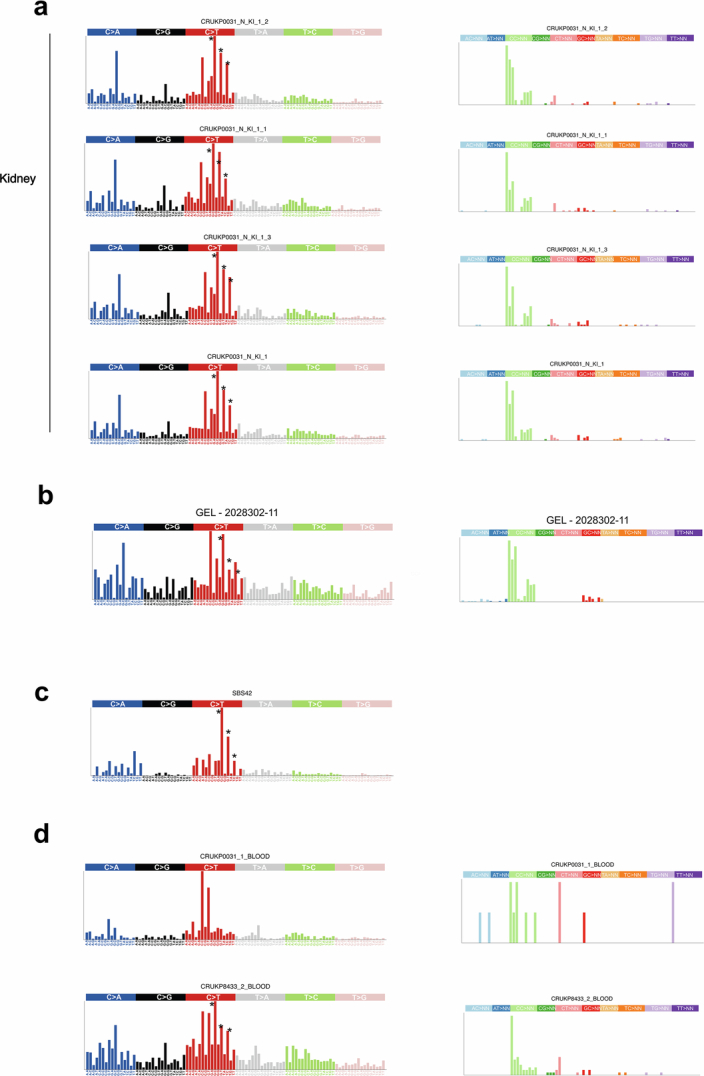
**A**. Single-base substitution profiles from 4 kidney samples from CRUKP0031, next to the corresponding double-base substitution (DBS) signature profiles. The defining mutational peaks of the SBS42 signature are highlighted as ‘*’ **B**. Mutational spectra of a Genomics England Cohort patient (GEL-2028302-11) diagnosed with kidney cancer, harbouring a cosine similarity >0.9 to the normal kidney samples from CRUKP0031. **C**. SBS42, related to haloalkane exposure. **D**. Top, the blood profile of CRUKP0031, revealing a clear cisplatin contribution. At right, the corresponding DBS pattern. Bottom, the blood profile of CRUKP8433. At right, the corresponding DBS pattern.

In samples from CRUKP0031, a 51-year-old woman who presented with collecting duct renal carcinoma, we identified 10 mutational signatures (Fig. 3g). This patient received a combined total of 16 cycles of cisplatin and carboplatin over a 4-year period, the most within the cohort. We observed that signatures SBS-D and SBS-C were pronounced in kidney samples from this patient (SBS-D median contribution = 33.51%, range 30.27–36.98%, SBS-C median contribution = 34.28, range 31.27–35.85%, *n* = 4), and these samples were characterized by higher SBS and DBS mutation rate per Mb per cell compared with those from other individuals (SBS median 1.44 versus SBS median 1.29 and DBS median 0.10 versus DBS median 0.02, respectively; Extended Data Fig. 6a). The DBS profile was characterized by CC>NN, resembling the profile of a clear cell renal cell carcinoma tumour analysed by whole-genome sequencing^23^ (Extended Data Fig. 6a,b). The SBS trinucleotide context profile of normal kidney samples from this patient was similar to SBS42 (average cosine similarity = 0.88; Extended Data Fig. 6c), a mutational signature linked to haloalkane exposure^33,34^. CRUKP0031 worked as a research chemist, raising the possibility that exposure to haloalkanes contributed to their mutational burden. However, the extended platinum treatment schedule of this patient coupled with the fact that we also observed SBS-D in a sample from patient CRUKP8433 (Extended Data Fig. 6d), who had no documented haloalkane exposure, may suggest that this is a mutational process related to platinum chemotherapy, which recapitulates the mutational effect of haloalkanes. Moreover, the strong correlation between SBS-D and SBS-C prevalence may indicate that these signatures are underpinned by a single process.

Together, these findings demonstrate how cancer therapies leave distinct and measurable mutational footprints on normal tissues that are influenced by the type and duration of treatment as well as the tissue context.

## Exogenous versus endogenous processes

**Fig. 4:**
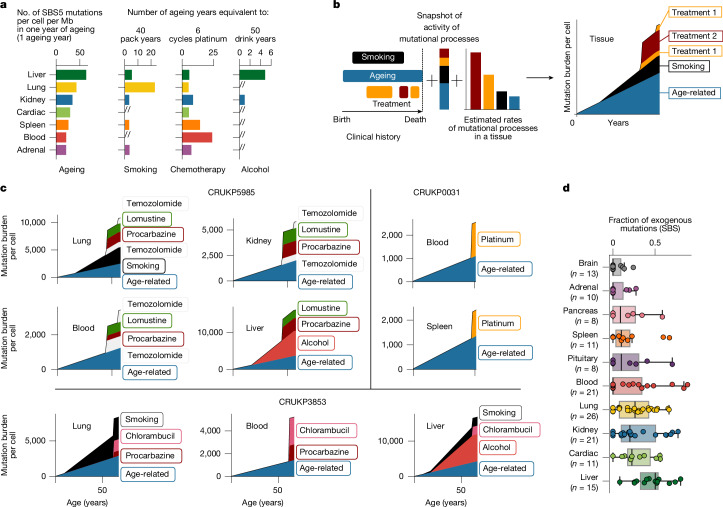
Relative effect of mutational processes. **a**, The relative effect of four different processes. Left to right: the number of SSB5 mutations per year of ageing in different tissues; the number of ageing years equivalent to 40 pack years; 6 cisplatin cycles; and 50 drink years in different tissues. **b**, Example mutation accumulation plots showing how different mutational processes may result in mutations in different tissues over time. **c**, Mutation accumulation plots across tissues from three individuals. **d**, Fraction of mutations attributed to exogenous mutational processes across tissues. Each dot represents one sample.

Finally, we compared and summarized the relative effect of different mutational processes in distinct tissues (Fig. 4a–c). In lung tissue, we estimated that a smoking history of 40 pack years would result in the same number of mutations as 20 years of ageing (that is, 20 ageing years), whereas 6 cycles of platinum chemotherapy was equivalent to 4.47 ageing years (Fig. 4a). By contrast, in blood, 6 cycles of platinum chemotherapy (the standard is typically 4–6), was estimated to cause an equivalent number of mutations to 27 ageing years (Fig. 4a).

Critically, whereas ageing, smoking and alcohol consumption are likely to lead to a gradual accumulation of mutations, treatment results in a punctuated burst of mutagenesis (Fig. 4b). For instance, in the blood of CRUKP0031, we estimated that there were 1,082 age-related mutations per cell, accumulated over the course of 51 years, whereas platinum chemotherapy-related mutagenesis yielded 1,420 mutations per cell during the course of treatment (Fig. 4c).

Taken together, less than 25% of mutations in samples from the brain could be attributed to damage induced from exogenous sources (Fig. 4d). Liver tissue was characterized by high exogenous mutational acquisition (IQR 33–53%), reflecting a combination of treatment-induced mutagenesis and DNA adducts probably caused by chemicals contained in alcohol (SBS-B) and tobacco smoke (SBS4).

## Selection in normal tissue

**Fig. 5:**
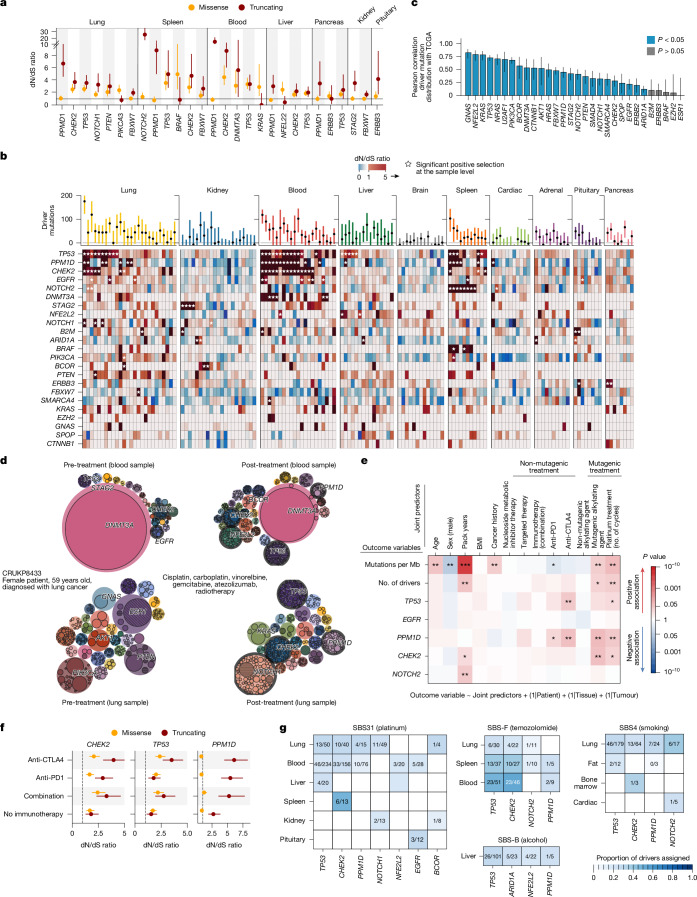
Selection in normal tissue. **a**, dN/dS analysis identifies genes exhibiting a significant excess of missense or truncating mutations across tissues within the cohort. Genes identified as significant after multiple testing correction are depicted (*q* < 0.2). **b**, dN/dS analysis on individual samples. Red indicates positive selection (dN/dS > 1) and blue (dN/dS < 1) indicates negative selection. A star denotes statistical significance (*q* < 0.2). **c**, Pearson’s *r* correlation between driver mutation prevalence in normal tissue versus TCGA (pan-cancer). Error bars depict 95% confidence intervals. **d**, Bubble plots illustrating gene-level selection for blood and lung samples from patient CRUKP8433 before (left) and following (right) treatment. For a given gene, each bubble reflects the relative cellular mutation rate based on the number of mutations, their VAFs and the number of sequenced bases. The outer circle is coloured if the gene is significantly mutated according to dNdScv. Individual bubbles inside a given gene bubble reflect the number of mutations and their type. **e**, Heat map indicating results from mixed-effects regression models. BMI, body mass index. **f**, dN/dS ratio for missense and nonsense mutations in *TP53*, *PPM1D* and *CHEK2*, with samples grouped by immunotherapy treatment (anti-CTLA4, 5 patients, 28 samples; anti-PD1, 4 patients, 44 samples; combination, 4 patients, 36 samples; no immunotherapy, 8 patients, 48 samples). Error bars denote 95% confidence intervals. **g**, The proportion of all driver mutations for each gene that can be attributed to the mutational signatures SBS31 (platinum), SBS-F (temozolomide), SBS-B (alcohol) and SBS4 (smoking). Only samples where the gene is deemed to be under positive selection were considered. The number of mutations corresponding to putative drivers is indicated as the numerator, with the denominator reflecting the total number of drivers for a given gene and sample type.

Next, to evaluate whether the mutations were sculpted by positive selection, we explored the ratio of nonsynonymous to synonymous mutation rates (dN/dS) (Fig. 5a,b). To account for both the different trinucleotide mutation rates resulting from distinct mutational processes and specific features of our duplex sequencing panel, we implemented dNdScv^35^ using a bespoke coverage-adjusted reference panel.

**Extended Data Fig. 7:**
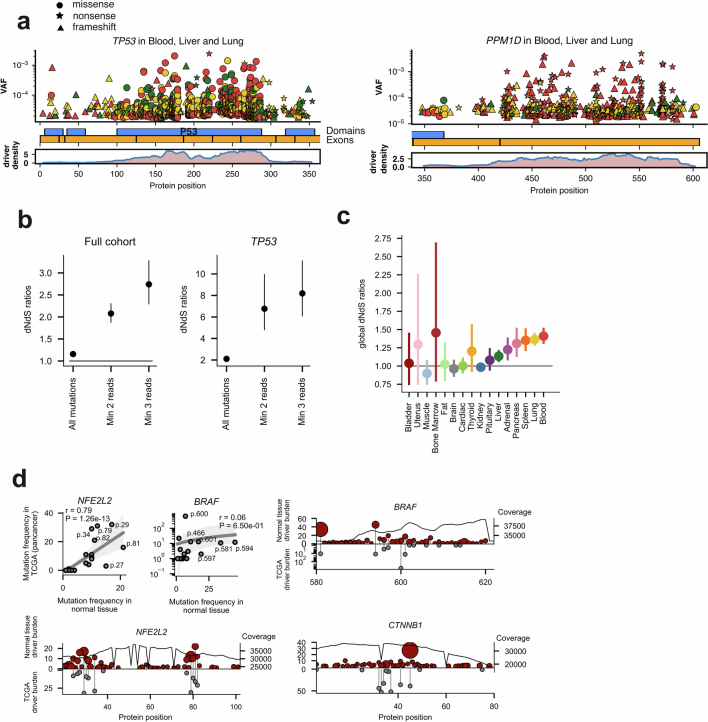
**A**. Distribution of observed driver mutations, as defined either by truncating, frameshift, or AlphaMissense score > 0.56, across the length of the *TP53* and *PPM1D* gene in blood, liver and lung tissues. Y axis represents the VAF of the observed mutations. The color represents the tissue, the shape highlights the consequence type of mutation. In blue, the protein domains as defined by pFam are depicted. *S*equenced exons are represented in orange, separated by black bars. At the bottom, the driver density across the gene body is depicted. **B** dNdS ratios for all mutations, those supported by at least 2 reads or supported by 3 reads, across the whole cohort or only in *TP53*. Lines represent the confidence intervals (CI). **C**. Global dNdScv rations across tissues. Lines represent the confidence intervals (CI). When the lower CI is above 1, we deem this tissue as being under global positive selection. **D**. Distribution of driver mutations in *NFE2L2*, *BRAF* and *CTNNB1*. The size of the dots represent the median VAF across mutated samples.

The gene with the most pervasive signal of positive selection across the cohort was *TP53*, which exhibited a significant excess of missense mutations in 34 out of 156 (21%) samples. Notably, in our cohort of 22 individuals, we observed a total of 2,439 missense mutations in *TP53* (Extended Data Fig. 7a). By comparison, a total of 2,314 missense mutations in *TP53* were identified in the 10,295 tumour samples available from the entire pan-cancer The Cancer Genome Atlas (TCGA) cohort^36^. However, although there was significant selection in *TP53*, we nevertheless identified 397 synonymous mutations, a considerably larger fraction compared with tumour tissues (75 synonymous mutations). This is consistent with the notion that we did not sample only large clonal expansions through duplex sequencing, but also many mutations in many small clones. Indeed, in keeping with this, the strength of selection was more pronounced in *TP53* and globally when we restricted our analysis to mutations with more than two variant counts (Extended Data Fig. 7b).

## Tissue-specific selection

We observed clear differences in selection between tissues and samples (Fig. 5a,b). When considered in aggregate, lung tissue exhibited evidence of selection across multiple cancer genes, including those linked to lung cancer such as *TP53*, *PTEN* and *PIK3CA* (Fig. 5a). At the sample level, we also observed normal samples with evidence of positive selection for *B2M* disruption and mutations in *EGFR* (Fig. 5b). In blood, we observed selection for mutations in genes previously associated with clonal haematopoiesis of indeterminate potential^37–39^, including in *CHEK2*, *DNMT3A* and *PPM1D*. Notably, however, the mutations in these genes were at VAFs significantly below the limit of detection using standard sequencing.

Certain tissues were characterized by an absence of significant gene mutations within our panel. For instance, samples derived from brain and cardiac tissue exhibited limited positive selection (Fig. 5a,b and Extended Data Fig. 7c).

Tissue samples from the spleen exhibited an enrichment for nonsense mutations in *NOTCH2* and missense mutations in *BRAF*, including mutations affecting D594 (*n* = 17). *NOTCH2* is a driver of splenic marginal zone lymphoma, whereas *BRAF*, and in particular *BRAF*^*V600E*^, is a known driver of hairy cell leukaemia. Hotspot analysis revealed a relative deficit of *BRAF* mutations affecting *BRAF*^*V600E*^ in the normal samples (Fig. 5c and Extended Data Fig. 7d), with a relative enrichment for p.591 and p.594 alterations. These mutations are not thought to activate the kinase activity of BRAF as strongly as the V600E mutation, and have therefore been labelled kinase-impaired^40^. Of note, in patient CRUKP7516 diagnosed with non-small cell lung cancer, we observed a significant excess of missense mutations in *BRAF* in normal tissues from both the lung and liver, as well as the spleen. In total, we identified 14 distinct individual putative *BRAF* driver mutations (AlphaMissense >0.56) across the 11 samples from this individual.

In liver samples, we observed a preponderance of missense mutations in *NFE2L2*, a known driver in hepatocellular carcinomas, including the hotspot mutations p.29 and p.81 (Extended Data Fig. 7d). Liver samples also exhibited preferential expansion and mutations in *CTNNB1*, which encodes β-catenin and is frequently subject to somatic alterations in hepatocellular carcinoma^41^. This included mutations in the S45 hotspot, an amino acid residue involved in phosphorylation and degradation of β-catenin, which is frequently mutated in liver cancers^42^ (Extended Data Fig. 7d).

**Extended Data Fig. 8:**
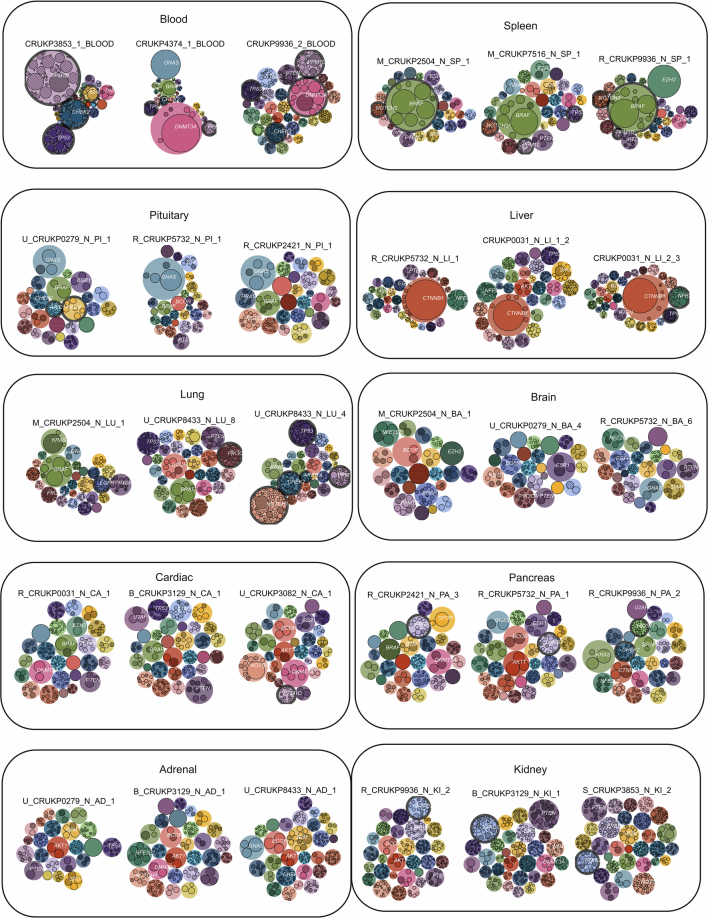
Representative bubble plots across the range of normal tissues within our cohort (see Methods).

Together, these data imply there are differences in selection pressures and the composition of clonal expansions across tissues (Extended Data Fig. 8).

## Effect of treatment on selection

**Extended Data Fig. 9:**
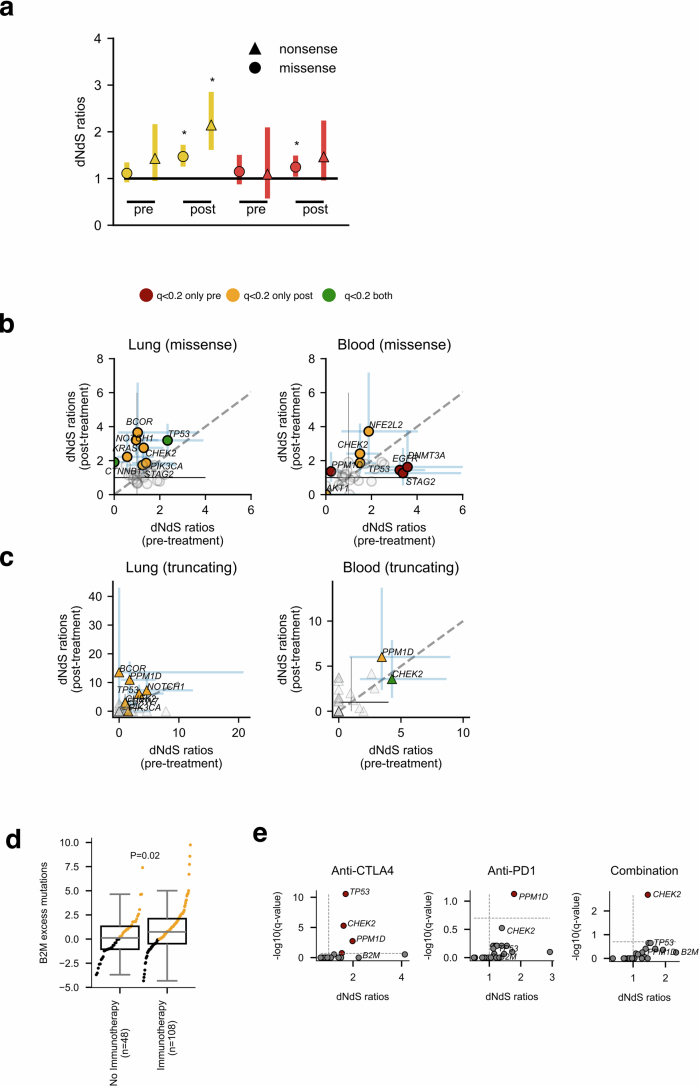
**A**. Global dNdScv rations for missense and nonsense mutations in pre-treated samples versus post-treated samples, in lung (yellow) and blood (red) tissues. Vertical lines represent the confidence intervals. When the lower boundary is higher than 1, we deem the set of mutations under positive selection. The plot shows that there is global positive selection in post-treated samples but not pre-treated samples. Lines represent the confidence intervals. **B**. Gene-wise (n = 30) correlation between dNdScv selection coefficients pre and post treatment, in blood and lung, across single-base substitutions. Lines represent the confidence intervals. Genes are deemed to be under selectionif q < 0.2 in dNdScv. **C**. Same as B, using indels if q < 0.2 in dNdScv. **C**. Same as B, using indels. **D**. Each dot reflects a normal tissue sample, the number of *B2M* mutations compared relative to expected (based on dNdScv). Samples are grouped according to whether they were administered immunotherapy. An excess of *B2M* mutations is observed in immunotherapy treated samples (P = 0.028, one-way wilcoxon test). **E**. Volcano plots showing in the x axis the maximum normalised ratio of dNdS values across samples from patients treated with different immunotherapies versus non immunotherapy treated, using variable_dNdS_twodatasets from Lawson et al.^7^ (Anti-CTLA4, 5 patients, 28 samples; Anti-PD1, 4 patients, 44 samples; Combination, 4 patients, 36 samples; No immunotherapy, 8 patients, 48 samples). Q-values are represented in the y axis.

To explore the effect of treatment on somatic selection in normal tissue, we first focussed on the paired samples taken before and following treatment with cisplatin. In lung and blood samples, we observed shifts in the patterns of clonal selection following treatment (Fig. 5d). For example, in the blood taken at the time of diagnosis in patient CRUKP8433 prior to treatment, we observed evidence for a large proportion of cells carrying a missense mutation in *DNMT3A*. However, following cisplatin treatment, we observed expansions of multiple clones in the blood, characterized by mutations in *TP53*, *PPMD1*, *CHEK2* and *NFE2L2*. In normal lung tissue prior to treatment, we observed clonal expansions associated with mutations in *PTEN* and *PIK3CA*. Following treatment with cisplatin, we observed selection for mutations in *CHEK2*, *PPMD1* and *TP53*. When considered together, we observed evidence for shifting patterns of selection following treatment (Extended Data Fig. 9a–c).

To investigate the effect of both mutagenic and non-mutagenic treatments, making use of the full cohort, we utilized multivariate mixed-effects models, incorporating our clinical and treatment information as covariates with mutation rates, and driver mutations as outcome variables. To control for the repeated sampling from individual patients and tissue differences, we also included ‘patient’, ‘tissue’ and ‘tumour type’ as mixed effects in the model (Fig. 5e).

As expected, we observed a significant positive relationship between mutation burden per cell and patient age (*P* = 3.07 × 10^−3^) as well as smoking burden (*P* = 4.45 × 10^−9^). Patients with a previous cancer history were associated with an increased mutation burden (*P* = 6.31 × 10^−3^). Mutation burden was also significantly positively associated with the number of cycles of platinum treatment (*P* = 0.0466), whether patients were administered mutagenic alkylating agents (*P* = 1.02 × 10^−3^), and negatively associated with the male sex (*P* = 2.32 × 10^−3^). Conversely, no significant positive increase in mutations was observed for immunotherapy treatments (anti-CTLA4: *P* = 0.14; anti-PD1: *P* = 0.049, with a negative association; combination treatment: *P* = 0.49), non-mutagenic alkylating agents (*P* = 0.28), nucleoside metabolic inhibitor therapy treatment (*P* = 0.79) or targeted therapy (*P* = 0.48).

In the context of cancer genes, we observed an increased prevalence of driver mutations in relation to smoking (*P* = 0.003915) as well as mutagenic treatments (mutagenic alkylating agents: *P* = 0.010; platinum treatment cycles: *P* = 0.00844). Smoking was specifically associated with driver mutations in *NOTCH2* (*P* = 0.00566). Driver mutations in *PPM1D* and *CHEK2* were significantly associated with platinum cycles (*PPM1D*: *P* = 0.0057; *CHEK2*: *P* = 0.015) and mutagenic alkylating (*PPM1D*: *P* = 0.0015; *CHEK2*: *P* = 0.0024) treatment.

In addition to an association with mutagenic treatment, we observed that *PPM1D* driver mutations were associated with immunotherapy treatment (anti-CTLA4: *P* = 0.0022; anti-PD1: *P* = 0.020; combination: *P* = 0.694) as were driver mutations in *TP53* (anti-CTLA4: *P* = 0.00957; anti-PD1: *P* = 0.1460; combination, *P* = 0.906). This suggests that immunotherapy, despite not directly inducing mutations, can act as a selection pressure, leading to selection for specific clones. Consistent with this, we also observed an increased preponderance of driver mutations in *B2M* in the context of immunotherapy in a univariate analysis (*P* = 0.02; Extended Data Fig. 9d). To further investigate the selection pressures associated with immunotherapy, we obtained dN/dS estimates for *TP53*, *CHEK2* and *PPM1D* in samples grouped by immunotherapy treatment status and observed a tendency for higher dN/dS values in immunotherapy treated samples (Fig. 5f). Likelihood ratio tests comparing the relative enrichment of nonsynonymous mutations in genes, while also correcting for differences in mutation rates, mutation signatures, coverage and selection at other genes, also identified *TP53*, *CHEK2* and *PPM1D* to be significantly associated with immunotherapy (Extended Data Fig. 9e), in keeping with the multivariate mixed-effects models.

**Extended Data Fig. 10:**
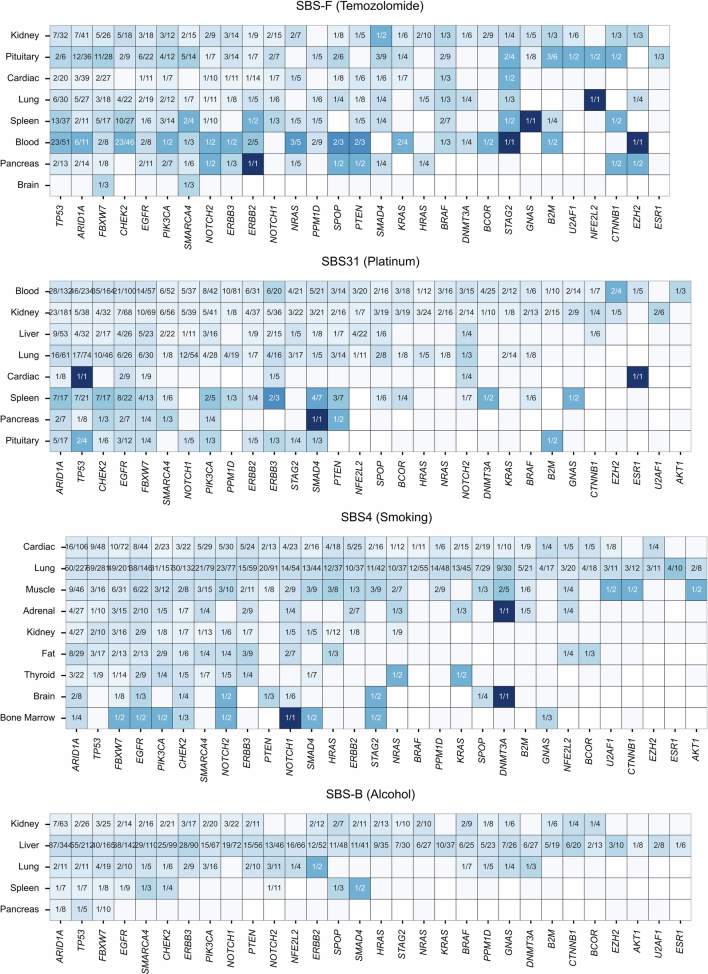
Same as Fig. 5h, but without restricting to genes under significant positive selection.

Next, to evaluate which treatments not only impose a selection pressure on normal tissue, but also directly induce mutations in cancer genes, we implemented a probabilistic approach to link putative driver mutations in genes under selection to the processes underpinning them (Fig. 5g). Focussing on samples from individuals receiving platinum chemotherapy, we observed evidence for treatment-induced driver mutations across different cancer genes within different tissues. For instance, in the context of *TP53*, 26% (13 out of 50) of *TP53* driver mutations in normal lung tissue, and 20% (46 out of 234) of *TP53* driver mutations in blood with detectable exposure to platinum treatment could be attributed to the platinum signature SBS31 (Fig. 5g). However, we observed limited evidence for platinum chemotherapy contributing to driver mutations in *PIK3CA* in lung tissue; this gene was not subject to significant positive selection in lung samples exposed to platinum and few putative driver mutations were generated (Extended Data Fig. 10).

In blood samples exhibiting detectable mutagenic exposure to temozolomide, 45% (23 out of 51) of driver mutations in *TP53* could be attributed to SBS-F, the temozolomide-related mutational signature (Fig. 5g). Similarly, 35% (13 out of 37) of *TP53* mutations in samples from the spleen could be linked to SBS-F. However, in lung samples, the effect of temozolomide treatment on the acquisition of *TP53* driver mutations was less profound, with 20% (6 out of 30) of *TP53* driver mutations linked to SBS-F.

In liver samples subject to platinum exposure, we observed that 20% (4 out of 20) and 18% (4 out of 22) of putative driver mutations in *TP53* and *NFE2L2*, respectively, could be linked to the platinum signature SBS31. By comparison, SBS-B, a mutational signature which correlates with alcohol exposure, could be linked to 25% (26 out of 101) of *TP53* and 18% (4 out of 22) of *NFE2L2* driver mutations (Fig. 5g).

It is worth noting that although treatment may provide an increased substrate for selection, this does not necessarily mean that most treatment-induced mutations are positively selected. For instance, we observed many treatment-induced mutations in *ARID1A*, yet limited evidence for positive selection of these mutations across the cohort (Fig. 5b,g and Extended Data Fig. 10).

Together, these data highlight how treatment can act both as a potent source of mutagens and as a selection pressure, sculpting somatic evolution in histologically normal tissue.

## Discussion

One in two people will be diagnosed with cancer during their lifetime^43^. Accordingly, the number of individuals who have been treated with cancer therapies is substantial and is likely to increase as cancers are detected earlier and novel therapies increase the chances of disease stabilization. Here we provide an analysis of the effect of diverse treatments and distinct environmental exposures on somatic evolution and mutation acquisition in 168 cancer-free samples from 16 tissues from 22 individuals.

Our results illustrate the profound effect of exogenous sources on somatic evolution in normal tissue and begin to shed light on how lifestyle choices and treatment decisions may affect future disease risk. In this cohort of heavily pre-treated patients with cancer, a median of 25% of mutations in each normal tissue sample could be attributed to exogenous factors. However, we observed clear differences in mutation acquisition and selection across tissues. For instance, although smoking contributed to somatic mutations in normal lung, cardiac, liver and kidney tissue, the rate of smoking-mediated mutagenesis was most pronounced in lung tissue, where we inferred that each pack year results in approximately 20 mutations per cell in the lung. Lung cancer risk is estimated to be approximately 25 times higher in men who smoke 15–24 cigarettes per day, compared with people who have never smoked^44^.

Similarly, although every tissue type investigated was subject to the mutagenic insults of platinum induced DNA damage, the highest rate of acquisition of platinum induced mutations was identified in blood, where 6 cycles of platinum chemotherapy generated an equivalent number of mutations as 25 years of ageing. By contrast, the same number of platinum cycles was equivalent to five years of ageing in the lungs. Platinum is commonly used in younger adults in the treatment of germ-cell testicular cancer and is associated with a hazard ratio of 2.4 for future solid malignancy risk. Notably, second solid tumour risk is dose-dependent, with high doses of platinum-containing chemotherapy (≥500 mg m^2^) being associated with a threefold increased lung cancer risk, compared with no significant increased risk associated with lower doses^45^. Consistent with this, we observed a dose-dependency of somatic mutation acquisition, with approximately 25 somatic mutations acquired per cell in the lung per cycle of platinum chemotherapy.

Nevertheless, it is notable that the risk of a future lung cancer following platinum treatment at a young age is tenfold lower than the risk associated with heavy smoking (relative risk 2.1 versus relative risk 25). Notwithstanding the extended period of smoking-mediated mutagenesis, this highlights that the effect of smoking on lung tissue is probably not solely restricted to its mutagenic effects, and that mutations alone are not sufficient to induce cancer^46^. Consistent with the importance of selective pressures that may not be mutagenic, we observed that immunotherapy was associated with selection for mutations in *TP53* and *PPM1D*. Further work may enable a deeper understanding of the complex interplay between different treatments and their mutagenic and non-mutagenic effects.

Although the high-depth duplex sequencing approach used in this study enabled somatic mutations to be captured at exceedingly low VAFs, we were not able to deconvolve the distinct cell types or cellular populations that were sequenced. Thus further work is warranted to disentangle and evaluate whether certain cell types in different tissues have a propensity to acquire mutations at an elevated rate. Indeed, recent work suggests that ageing and treatment may substantially alter the architecture of blood cell populations^28^.

Over millions of years, species have evolved protective mechanisms to keep the incidence of cancer low, including high-fidelity DNA replication and multiple DNA repair processes. However, selection declines following reproductive age. Moreover, humans have not adapted or evolved processes to limit the effect of exposure to mutagenic and non-mutagenic insults from, for example, environmental exposures, tobacco smoke, cytotoxic chemotherapies or immunotherapies. Further work, including systematic surveys and mechanistic studies, is required to quantify and assess the effect of environmental insults and treatment-induced mutagenesis on distinct normal cell types and how this relates to somatic evolution, premature ageing and the development of diseases, including cancer.

## Methods

### Tissue collection and sample preparation

Tissue was collected from patients enroled in the PEACE study (ethics approval reference 11/LO/1996).

Samples were selected from organs where no metastasis was evident during autopsy. Patients were prioritized according to the number of different organ sites available for collection, and to enable balanced sex and mixed age representation. Samples were collected from anatomical regions, snap frozen in liquid nitrogen and stored long term in the −80 °C freezer. All samples (*n* = 168) used in the cohort lockdown were bioinformatically assessed for presence of infiltrating cancer cells and large-scale allelic imbalance (see below) and deemed cancer-free. Additionally, for 118 cases, we performed pathology review to provide additional evidence of cancer-free status. Pathology review involved analysis of adjacent tissue to the sequenced samples that were fixed in formalin, embedded in paraffin and stained with haematoxylin and eosin before scanning with a NanoZoomer digital pathology system (Hamamatsu). Digital slides were then examined to evaluate the absence or presence of malignancy. This revealed 18 samples where adjacent tissue to the sequenced tissue either contained tumour cells or could not be confidently classified as cancer-free. Removing these samples, which were bioinformatically defined as cancer-free, did not qualitatively alter any of the results. Additionally, five metastatic samples from five different patients were collected. A 2 mm^3^ piece of tissue was processed for DNA extraction using the Qiagen AllPrep kit following the manufacturer’s instructions. DNA from blood was purified using the DNeasy Blood and Tissue kit (Qiagen). Purified nucleic acids were accessed for yield and purity using DNA Broad Range assay kits (Invitrogen).

### Panel design

To investigate mutational processes representative of genome-wide trinucleotide content, we designed a targeted genomic panel spanning 82.5 kb focusing on 30 cancer and normal tissue driver gene regions, selected taking into account the mutation frequency in multiple cancer and normal tissue cohorts^14^. The regions included in our panel are detailed in Supplementary Table 2. In addition to the driver gene regions, our panel also encompasses several genomic regions with comparable representation of the genome-wide trinucleotide context that are under neutral selection, as defined by Twinstrand Bioscience. These regions are used to study the mutational processes in a context that is not influenced by selective pressures.

### Library preparation and sequencing

Libraries were prepared using 1,000 ng of extracted gDNA as input into the TwinStrand DuplexSeq Library Preparation Kit as per the manufacturer’s guidelines. In brief, 1,000 ng of gDNA underwent enzymatic fragmentation, end repair and A-tailing before being ligated with unique DuplexSeq adapters followed by 10 cycles of an indexing PCR reaction. Hybrid capture was then performed using a custom 82.5-kb capture panel from TwinStrand, followed by 16 cycles of PCR amplification. Libraries underwent a second round of hybridization using the same custom capture panel, followed by another five cycles of PCR amplification. The final libraries were then quantified and assessed using the Qubit fluorometer (Thermo Fisher Scientific) and TapeStation 4200 (Agilent) before being sequenced with 150 bp paired end reads on the Illumina NovaSeq 6000 system.

### NanoSeq libraries

Libraries were prepared using the NanoSeq protocol as previously described^16^. In brief, 2 ng of extraction gDNA was purified using a 1:1 mixture of nuclease-free water and SPRIselect beads (Beckman Coulter, B23319). Samples were then fragmented on-bead using the HpyCH4V restriction enzyme (New England Biolabs, R0620S) at 37 °C for 15 min and purified with 2.5× SPRIselect beads. The fragmented and cleaned up DNA was A-tailed and ligated with xGen CS Duplex Adapters (Integrated DNA Technologies, 1080799) and purified again with 1× SPRIselect beads, resuspending in a final volume of 20 μl nuclease-free water.

The adapter ligated libraries were quantified by quantitative PCR (qPCR) using the KAPA Library Quantification Kit (Roche, KK4828) with custom primers, as previously described^16^. Using the qPCR concentrations, libraries were normalized to 0.6 fmol in 20 μl nuclease-free water. The normalized libraries were added to the PCR mastermix containing 25 μl NEBNext Ultra II Q5 Master Mix (New England Biolabs, M0544S) and 5 μl xGen UDI Primers (Integrated DNA Technologies, 10008052). Libraries were amplified for a total of 13 cycles and cleaned up twice using 0.7× SPRIselect beads. The final libraries were assessed using the Qubit fluorometer (Thermo Fisher, Q33231) and Tapestation 4200 D1000 Assay (Agilent, 5067–5582). Libraries were then pooled and sequenced using 150 bp paired end reads on the Illumina NovaSeq 6000 platform, aiming for 30× coverage per sample.

### Non-duplex NanoSeq libraries

Non-duplex NanoSeq libraries were prepared using the protocol as described above, with the following modifications of 10 ng of input DNA into the library preparation. Libraries were not quantified by qPCR but instead the entire volume of library was used for a 10-cycle indexing PCR. The remainder of the protocol was carried out as described above.

### Duplex sequencing bioinformatic analyses

Duplex sequencing fastqs were processed using a bespoke Nextflow^47^ pipeline that uses the fgbio suite (v.2.2.1) and follows the best practices for duplex sequencing processing (https://github.com/oriolpich/normal_tissues_nature_2025/tree/main/src/duplex_nf/DuplexPipe). In brief, FastqToBam was used to extract unique molecular identifiers, followed by alignment using bwa-mem (v.0.7.17)^48^. The reference genome used was GRCh38 (RefSeq assembly GCA_000001405.15), using a version without alternative contigs (no_alt_analysis_set), with an applied patching to allow proper mapping of the *U2FA1* gene.

The bams were reformatted and template-coordinate sorted using ZipperBam and samtools (v.1.19.2) respectively. We then used GroupReadsByUmi (–edits = 1, min-map-q = 10, strategy=paired), and called consensus reads using CallDuplexConsensusReads, with minimum four consensus reads (at least two for each strand) (min-reads = 4 2 2, error_rate_pre_umi = 45, error_rate_post_umi = 40, min_input_base_quality = 20). The consensus reads were then remapped to the reference genome, and the bam containing the consensus reads aligned was filtered using FilterConsensusReads (--max-read-error-rate = 0.025 --max-base-error-rate = 0.05 --min-mean-base-quality = 50 --min-base-quality = 60 --max-no-call-fraction = 0.2 --require-single-strand-agreement true). Reads were then hard-clipped (ClipBam, --read-one-five-prime 7 --read-two-five-prime 7) and finally reads were removed if the minimum difference between the primary alignment score and the secondary alignments in forward and reverse^16^ was less than 50 (AS – XS ≤ 50).

Mosdepth (v.0.3.5)^49^ was used to identify base-pair coverage and in-target coverage. We included off-target regions (that is, regions that we did not cover originally with our panel), if the median coverage was higher than 15,000. We then called variants using VarDict^50^ in these regions.

Point mutations were excluded if any of the following applied: (1) the number of mismatches in reads supporting the variant was greater than 4; (2) the proportion of Ns at the mutated position (bases with no consensus) was greater than 0.05; or (3) the coverage was lower than the sample median depth minus three times the depth standard deviation. For indels, variants were removed when the proportion of Ns was greater than 0.10 or when they failed the same coverage filter.

We applied a Kolmogorov–Smirnov test to remove recurrent artefacts based on the distribution of mutant base positions within reads^51^ (false discovery rate (FDR) < 0.05). We also applied a whole-genome single nucleotide polymorphism (SNP) mask that comprises common SNPs, and the NOISE mask that contains sites with elevated error rates, from Abascal et al.^16^, removed variants with mean mismatches greater than 4, and discarded indels with MSI >5 as annotated by VarDict. To filter putative cross-sample contaminants, we computed a *P* value for each variant observed more than 20 times in the cohort under a global binomial model parameterized by the cohort aggregate VAF; variants with FDR < 0.05 were removed. Bona fide driver hotspots defined by Chang et al.^52^ supplemented with 2 driver indel hotspots (chr17_60663262_CA_C [PPM1D] and chr1_26779439_TG_T [ARID1A]), were exempt from this contamination filter.

All sites failing any of these filters, except those that only failed the Kolmogorov–Smirnov test, were aggregated into a site blacklist, which was subsequently used for analyses of positive selection.

Mutations were further classified as probably coming from blood samples using a conservative approach. For each patient, mutations are then classified as blood-like if they are found in both blood and more than 25% of non-blood samples (unless they are bona fide *EGFR*, *PIK3CA* or *KRAS* driver hotspots, in which case they are labelled as suspicious), or suspicious if found in more than 50% of non-blood samples but not in blood. For patients without blood samples, mutations are classified as blood-like if they occur in more than 50% of samples. Mutations deemed as non-blood-like were used for positive selection analyses.

Mutations were then annotated using Variant Ensembl Predictor (v.109)^53^. Variants were also annotated with AlphaMissense^54^, and mutations with a score higher than 0.56 were deemed as putative driver mutations, as described in the original publication.

Nanoseq samples were processed as described in their original publication (https://github.com/cancerit/NanoSeq, release 3.5.5). The normal control was either a whole-genome sequenced sample or another NanoSeq sequenced sample from the same patient.

### Identification of SNPs

Homozygous SNPs were identified based on having a VAF greater than 0.95 in all samples from an individual patient. Putative heterozygous SNPs were identified as any variant present in all samples with a VAF between 0.01-0.85.

### Identification of somatic copy number alterations and potential tumour contamination in individual samples

To explore the presence of somatic copy number alterations in individual samples we considered whether heterozygous SNPs deviated from the expected 0.5 or the median value across samples (thus taking into account any alignment biases).

To test for deviation, we employed a two-sided binomial test in R using the prop.test function. For each sample we calculated the proportion of SNPs that deviated from 0.5 and also the proportion of SNPs that deviated from the median proportion across samples. We observed that tumour samples exhibited clear deviation from expected values, with evidence of somatic copy number alterations associated with clonal expansions. Samples that exhibited any evidence for clonal expansion of somatic copy number alteration were removed. While these somatic copy number alterations may reflect non-malignant clonal expansions, we excluded these samples as we could not rule out potential malignant infiltration.

To further assess potential cancer cell infiltration into normal tissue we considered whether heterozygous SNPs deviated consistently between samples, potentially indicating shared clones or tumour contamination. In brief, for individuals where we sequenced a tumour sample (or a sample with a clonal expansion), we identified all heterozygous SNPs in the sample that exhibited evidence for allelic imbalance (*P* < 0.05, binomial test). We divided this set of SNPs into two groups, ‘high’ or ‘low’, reflecting whether the B-allele frequency was significantly greater than the median or not. Any normal sample with contamination would be expected to exhibit a significant bias whereby SNPs in the ‘high’ category should have a higher B-allele frequency than those in the ‘low’ category. We therefore performed a one-way Wilcoxon test to evaluate contamination. The following samples were removed as a result of potential contamination: CRUKP5732_2_BLOOD, R_CRUKP5732_N_AD_1, R_CRUKP5732_N_CA_1 and R_CRUKP0031_N_AD_1. Finally, we clustered samples from each individual based on the cosine similarity of their B allele frequencies. We removed any samples that clustered together with sequenced tumour samples. This led to the exclusion of R_CRUKP0031_N_LI_2, CRUKP0031_N_LI_2_1 and CRUKP0031_N_LI_2_2.

### Mutational signature analysis

Hierarchical Dirichlet processes (HDP), available at https://github.com/nicolaroberts/hdp, was run to extract mutational signatures across 100 independent chains with two layers, the first one patient and the second one tissue.

For SBS extraction, we used ‘SBS1’, ‘SBS5’, ‘SBS40’, ‘SBS4’, ‘SBS92’, ‘SBS25’, ‘SBS31’, ‘SBS35’ and ‘SBS17b’ from COSMIC v.3.4 and ‘Temozolomide’ from Kucab et al.^27^, as priors, with the following parameters: n_posterior **=** 100, n_space **=** 2000, nburnin **=** 500000, ninitial_clust **=** 25, prior_c = 1000. For indels extraction, we used all COSMIC indels as priors and included the radiotherapy-related signature described in ref. ^29^, and run HDP with the following parameters: n_posterior **=** 200,n_space **=** 2000, nburnin **=** 50000, ninitial_clust **=** 30, prior_c = 20000. For double-base substition extraction, we used all COSMIC DBS signatures as priors, plus DeGasperi DBS related to cisplatin^23^. HDP was then run with the following parameters: n_posterior **=** 200, n_space **=** 2000, nburnin **=** 50000, ninitial_clust **=** 30, prior_c = 20000.

All of the extracted signatures were matched to COSMIC if the cosine similarity was higher than 0.9. Otherwise, we named each of the non-COSMIC signatures alphabetically (SBS-A, SBS-B, and so on).

The signature attribution to individual mutations was done as previously described^13,55^. In brief, given a set of exposures to mutational signatures and their mutational profile, a probability per type of mutation per patient can be generated.

### Mutations per genome per cell

As discussed^5,11^, the mutations per genome per cell (*β*) can be approximated as:

$$\beta \approx 2\times \sum _{j}({\mathrm{VAF}}_{j})/{L}_{\mathrm{Mb}}$$

Where *j* is each observed mutation, and *L*_Mb_ is the number of megabases sequenced with good enough coverage from our panel. The attribution of mutations per genome per cell to each signature was performed by multiplying the value to each of the signature exposures.

### Relative effect of mutational processes

The contribution of certain mutational processes with respect to age was obtained by dividing their slopes. This value is scaled by 40 in smoking (considered a heavy smoker), 6 in platinum (average cycles) and 50 (average drinking years). In Fig. 4c, for treatments where we could not calculate the slope, we divided the number of treatment-related mutations and divided by the start and end date of treatment.

### TCGA and other cohorts

TCGA PanCanAtlas data was obtained from ref. ^36^. In order to calculate the number of mutations per genome of the most common recent ancestor, we applied an approach to derive clonal and subclonal mutations^56^. We then selected the clonal mutations, and normalized by the length of the exome. Mutations per genome in datasets from refs. ^3,4^ were calculated using the same formula as above, utilizing the size of the covered region as defined in the papers.

### Positive selection analyses

To evaluate positive and negative selection we implemented dNdScv^35^ using a bespoke reference coding sequence reflecting our sequencing panel. To mitigate against varied coverage across our panel, for each individual sample, we modified the mutation opportunity matrix *L* to reflect the sequence coverage. In brief, we obtained a coverage bed file for each sample, and then for each gene obtained the median coverage at each of the 192 channels, and adjusted the *L* matrix accordingly. Blacklisted sites were given a coverage of 0. We did the same for each tissue and for the full cohort for specific analyses.

For each sample and each gene within our panel we obtained dNdScv missense and nonsense values. To avoid depicting spurious dN/dS values resulting from genes and samples with few mutations, in Fig. 5b we additionally filtered genes in relation to the number expected missense, nonsense and silent mutations.

Specifically, in the context of missense dNdScv values, we filtered gene:sample combinations that exhibited:

<4 expected and <3 observed missense variants, or <2 total observed variants.

Likewise, in the context of nonsense dNdScv values, we filtered gene:sample combinations that exhibited:

<2 expected and observed nonsense variants, or if the combined synonymous and nonsense expected variants was <3 with <2 observed events.

Drivers were then identified using ‘qglobalpos_cv’<0.1 | ‘qsubpos_cv’<0.05 | ‘qindpos_cv’ <0.05.

### Radiotherapy exposure assessment

The likelihood of a given tissue being exposed to radiotherapy was assessed by a trained radiologist using the clinical data of each patient, blinded to the radiotherapy-indel signature exposures.

### Coverage and variance explained

The relationship between coverage, VAF and number of mutations was assessed through a linear mixed-effects model using the R package lme4 followed by an anova test.

### Linear mixed-effects models

To evaluate the effect of both mutagenic and non-mutagenic treatment agents on different outcome variables we performed mixed-effects multivariable regression analysis. We focussed on a selection of key cancer genes (the five genes with the most excess mutations across samples), in addition to the total number of drivers, as well as mutation burden per cell. To control for the repeated samples from patients, the range of tissues and the distinct tumours which these patients harboured, we also include ‘patient’, ‘tissue’ and ‘tumour type’ as mixed effects in the model, using the lmerTest package in R.

### Pack years and drink years

To estimate lifetime exposure to cigarettes we used pack years; calculated by multiplying the number of packs of cigarettes smoked per day (self-reported) by the number of years a person has smoked (self-reported). For example, smoking one pack (20 cigarettes) a day for 20 years equals 20 pack years, and smoking two packs (40 cigarettes) a day for 10 years also equals 20 pack years. We derived a similar metric to estimate lifetime alcohol consumption, ‘drink years’. We defined a baseline ‘drink year’ as drinking 14 units of alcohol a week for a year. For reference, this equates to drinking 6 pints (568 ml) of standard strength beer (5% ABV) a week for a year. Given that we did not have historical drinking information (for example, when the individual started drinking), we make the assumption that drinking is approximately consistent from the age of 18.

### Reporting summary

Further information on research design is available in the Nature Portfolio Reporting Summary linked to this article.

## Online content

Any methods, additional references, Nature Portfolio reporting summaries, source data, extended data, supplementary information, acknowledgements, peer review information; details of author contributions and competing interests; and statements of data and code availability are available at 10.1038/s41586-025-09792-4.

## Supplementary information

Supplementary Note Supplementary Note, including one figure.

Reporting Summary

Supplementary Tables This file contains Supplementary Tables 1–5. Supplementary Table 1: patients and clinical characteristics. Supplementary Table 2: bed file with the bespoke panel. Supplementary Table 3: somatic mutations. Supplementary Table 4: mutational signatures. Supplementary Table 5: samples used in the study.

## Data Availability

Duplex-seq, Nanoseq and whole-genome sequencing data are available in FASTQs at the European Genome–Phenome Archive (EGA) with the identifier EGAD00001015726. Data are available through the Cancer Research UK and University College London Cancer Trials Centre (ctc.peace@ucl.ac.uk) for academic, non-commercial research purposes upon reasonable request and subject to review of a project proposal that will be evaluated by a PEACE data access committee, entering into an appropriate data access agreement and subject to any applicable ethical approvals.

## Code availability

All analyses were performed using Python v.3.7 and R v.4.1. The full analysis pipeline and all code required to reproduce the results are available at https://github.com/oriolpich/normal_tissues_nature_2025/.
