# Supplementary material for: Somatic evolution following cancer treatment in normal tissue
Source: Nature. 2025 Dec 10;653(8115):900–10. doi: 10.1038/s41586-025-09792-4 (PMC13190248; doi:10.1038/s41586-025-09792-4)
Supplement: Supplementary file 1 — Supplementary Note, including one figure. [file 41586_2025_9792_MOESM1_ESM.pdf]

nature portfolio

https://doi.org/10.1038/s41586-025-09792-4

Supplementary information

Somatic evolution following cancer treatment in normal tissue

In the format provided by the authors and unedited

Supplementary Note

1. Error rate comparison between Twinstrand and NanoSeq

Previous work (Lawson et al., 2024) has estimated that duplex sequencing, with enzymatic fragmentation which is the same methodology we use, yields errors around 4×10^{-8} errors/bp. Critically, if our mutation burden is similar to the estimated error rate, it would be difficult to distinguish any real mutations from errors. Therefore, we have added a number of additional analyses, including comparison of our mutation burden to the error rate, and second, orthogonal validation using NanoSeq.

We can estimate our mutation frequency (M) using the sample-specific duplex median coverage C, and the length of our panel, L:

$$\text{Total Base Pairs Sequenced} = L\ (\text{bp}) \times C$$
$$\text{Mutation Frequency} = \frac{M}{L \times C}$$

The median mutation frequency we observe is $3.73e^{-07}$, which is significantly higher than the experimental error rate. This suggests that our mutations cannot be explained by sequencing artefacts (**Supplementary Note Figure 1a)**.

2. *Application of NanoSeq to our cohort.*

To further validate our approach and ensure additional confidence in our results, we have also subjected 27 samples to NanoSeq (Abascal et al, 2021), a state-of-the-art duplex sequencing approach. As a control, either another sample from the same patient was used as a control, or a non-duplex whole-genome sequenced sample.

The efficiency of the protocol lies within optimal ranges and is comparable to the original NanoSeq publication (Abascal et al, 2021, Extended Data Figure 4 in their manuscript (**Supplementary Note Figure 1b)**.

To assess whether we obtain concordant mutation burden estimates, we correlated the corrected mutation burden from the NanoSeq pipeline to our estimated mutations per cell per megabase. This revealed a statistically significant correlation between the two (Pearson's $r = 0.87$, $P = 2.7e-06$) (**Supplementary Note Figure 1c)**.

To further validate the mutations obtained using our Twinstrand duplex sequencing approach, we performed a comparison of mutational profiles. We observed that 96-based trinucleotide profiles generated using the orthogonal technologies exhibited a high consistency (see Figure

below as an example). This suggests the mutations reflect real biological processes (**Supplementary Note Figure 1d)**.

The mean cosine similarity when the same samples are considered is 0.94, whereas the mean cosine similarity between samples from the same tissue is 0.87, and random profiles from other tissues is 0.46 (**Supplementary Note Figure 1e**).

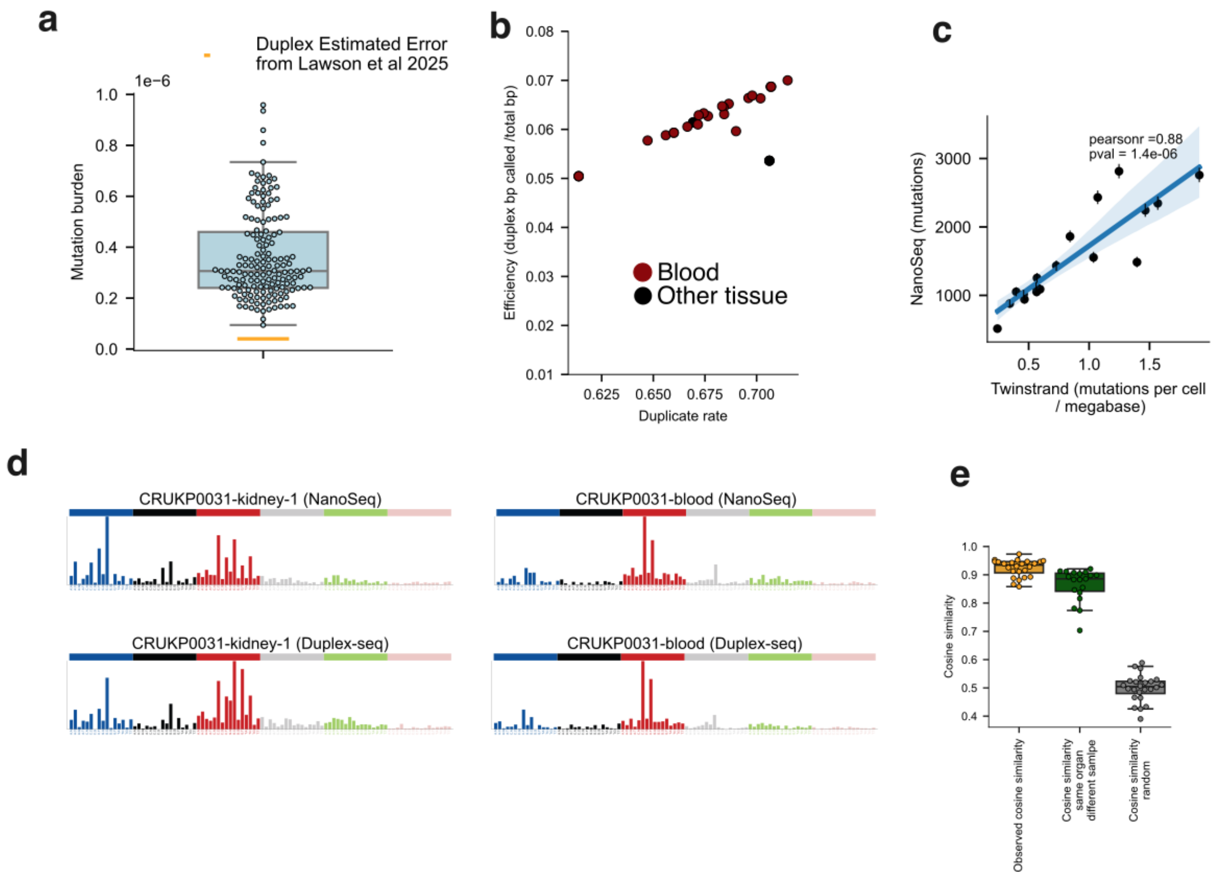

Supplementary Note Figure. a) Distribution of the SNV mutation burden across all of the samples within our study, and the estimated duplex error rate from Lawson et al, 2024. **b)** Left, the estimated duplex efficiency in our processed samples across blood and other tissues, comparable to what the authors report in their original publication on the right hindsight. **c)** Positive correlation between our mutations per cell/megabase measurements and NanoSeq corrected whole-genome corrected estimates. **d)** Two samples CRUKP0031-blood and CRUKP0031-kidney-1, profiled with NanoSeq technology and Twinstrand Duplex-seq technology. The mutational spectra have a cosine similarity of 0.97 and 0.96, respectively. **e)** Distribution of cosine similarities between NanoSeq sequenced samples and twinstrand sequenced samples across three different scenarios, the first one when comparing the same sample, the second when comparing other samples from the same tissue, and the third one when comparing the profile to random profiles.
