# Supplementary material for: Somatic evolution following cancer treatment in normal tissue
Source: Nature. 2025 Dec 10;653(8115):900–10. doi: 10.1038/s41586-025-09792-4 (PMC13190248; doi:10.1038/s41586-025-09792-4)
Supplement: Supplementary file 2 — Reporting Summary [file 41586_2025_9792_MOESM2_ESM.pdf]

nature portfolio

Corresponding author(s): Oriol Pich

Last updated by author(s): Oct 17, 2025

Reporting Summary

Nature Portfolio wishes to improve the reproducibility of the work that we publish. This form provides structure for consistency and transparency in reporting. For further information on Nature Portfolio policies, see our Editorial Policies and the Editorial Policy Checklist.

Statistics

For all statistical analyses, confirm that the following items are present in the figure legend, table legend, main text, or Methods section.

n/a	Confirmed	
☐	☒	The exact sample size (*n*) for each experimental group/condition, given as a discrete number and unit of measurement
☐	☒	A statement on whether measurements were taken from distinct samples or whether the same sample was measured repeatedly
☐	☒	The statistical test(s) used AND whether they are one- or two-sided<br>*Only common tests should be described solely by name; describe more complex techniques in the Methods section.*
☐	☒	A description of all covariates tested
☐	☒	A description of any assumptions or corrections, such as tests of normality and adjustment for multiple comparisons
☐	☒	A full description of the statistical parameters including central tendency (e.g. means) or other basic estimates (e.g. regression coefficient) AND variation (e.g. standard deviation) or associated estimates of uncertainty (e.g. confidence intervals)
☐	☒	For null hypothesis testing, the test statistic (e.g. *F*, *t*, *r*) with confidence intervals, effect sizes, degrees of freedom and *P* value noted<br>*Give P values as exact values whenever suitable.*
☒	☐	For Bayesian analysis, information on the choice of priors and Markov chain Monte Carlo settings
☒	☐	For hierarchical and complex designs, identification of the appropriate level for tests and full reporting of outcomes
☒	☐	Estimates of effect sizes (e.g. Cohen's *d*, Pearson's *r*), indicating how they were calculated

Our web collection on statistics for biologists contains articles on many of the points above.

Software and code

Policy information about availability of computer code

Data collection	N/A
Data analysis	All code to reproduce the analyses and the necessary software can be obtained here<br>https://github.com/oriolpich/normal_tissues_nature_2025/

For manuscripts utilizing custom algorithms or software that are central to the research but not yet described in published literature, software must be made available to editors and reviewers. We strongly encourage code deposition in a community repository (e.g. GitHub). See the Nature Portfolio guidelines for submitting code & software for further information.

Data

Policy information about availability of data

All manuscripts must include a data availability statement. This statement should provide the following information, where applicable:

- Accession codes, unique identifiers, or web links for publicly available datasets
- A description of any restrictions on data availability
- For clinical datasets or third party data, please ensure that the statement adheres to our policy

Raw data from PEACE patients analyzed in this study including fastq will be deposited at the European Genome–phenome Archive (EGA), hosted by The European Bioinformatics Institute (EBI) and the Centre for Genomic Regulation (CRG) under accession codes (EGAS00001006494) under controlled access.

Research involving human participants, their data, or biological material

Policy information about studies with human participants or human data. See also policy information about sex, gender (identity/presentation), and sexual orientation and race, ethnicity and racism.

Reporting on sex and gender	Sex information has been included in the data shared online.
Reporting on race, ethnicity, or other socially relevant groupings	Information on race, ethicity or other socially relevant groupings was not included in the manuscript.
Population characteristics	Cohort demographics (n =22) are included in Extended Data Table 1, and further patient-level details are available with supporting molecular data on Zenodo.
Recruitment	When patients are initially diagnosed with stage I-III lung cancer and then referred for surgical resection, a research nurse identifies them on a clinic/operating list. The patient has an initial eligibility assessment and is then provided with written information about the TRACERx study and he/she can ask the research nurse any questions.
Ethics oversight	This study was approved by the NRES Committee London with the following details:<br>Study title: TRAcking non small cell lung Cancer Evolution through therapy (Rx)<br>REC reference: 13/LO/1546<br>Protocol number: UCL/12/0279<br>IRAS project ID: 138871<br>Written informed consent was obtained from all participants.

Note that full information on the approval of the study protocol must also be provided in the manuscript.

Field-specific reporting

Please select the one below that is the best fit for your research. If you are not sure, read the appropriate sections before making your selection.

☒ Life sciences ☐ Behavioural & social sciences ☐ Ecological, evolutionary & environmental sciences

For a reference copy of the document with all sections, see nature.com/documents/nr-reporting-summary-flat.pdf

Life sciences study design

All studies must disclose on these points even when the disclosure is negative.

Sample size	Here, we report analyses from normal tissues across 22 patients. No sample size calculations were performed
Data exclusions	N/A
Replication	N/A
Randomization	N/A
Blinding	Blinding to mutational signature exposures was performed when a clinical oncologist assessed the likely impact of radiotherapy across each patient tissues.

Reporting for specific materials, systems and methods

We require information from authors about some types of materials, experimental systems and methods used in many studies. Here, indicate whether each material, system or method listed is relevant to your study. If you are not sure if a list item applies to your research, read the appropriate section before selecting a response.

Materials & experimental systems

n/a	Involved in the study
☒	☐ Antibodies
☒	☐ Eukaryotic cell lines
☒	☐ Palaeontology and archaeology
☒	☐ Animals and other organisms
☐	☒ Clinical data
☒	☐ Dual use research of concern
☒	☐ Plants

Methods

n/a	Involved in the study
☒	☐ ChIP-seq
☒	☐ Flow cytometry
☒	☐ MRI-based neuroimaging

Clinical data

Policy information about clinical studies

All manuscripts should comply with the ICMJE guidelines for publication of clinical research and a completed CONSORT checklist must be included with all submissions.

Clinical trial registration: PEACE (Posthumous Evaluation of Advanced Cancer Environment; NCT03004755

Study protocol: https://clinicaltrials.gov/study/NCT03004755

Data collection: Clinical and pathological data is collected from patients during study onset. Data collection is overseen by the sponsor of the study (Cancer Research UK & UCL Cancer Trials Centre) and takes place in hospitals across the United Kingdom. A centralised database called MACRO is used for this purpose.

Outcomes: PEACE (Posthumous Evaluation of Advanced Cancer Environment; NCT03004755) is a national, multicentre, pan-cancer research autopsy programme. For two patients, in addition to obtaining cancer-free tissue from autopsy, normal tissue samples collected prior to any cancer treatment were available through the TRACERx study (ClinicalTrials.gov identifier: NCT01888601)

Plants

Seed stocks: *Report on the source of all seed stocks or other plant material used. If applicable, state the seed stock centre and catalogue number. If plant specimens were collected from the field, describe the collection location, date and sampling procedures.*

Novel plant genotypes: *Describe the methods by which all novel plant genotypes were produced. This includes those generated by transgenic approaches, gene editing, chemical/radiation-based mutagenesis and hybridization. For transgenic lines, describe the transformation method, the number of independent lines analyzed and the generation upon which experiments were performed. For gene-edited lines, describe the editor used, the endogenous sequence targeted for editing, the targeting guide RNA sequence (if applicable) and how the editor was applied.*

Authentication: *Describe any authentication procedures for each seed stock used or novel genotype generated. Describe any experiments used to assess the effect of a mutation and, where applicable, how potential secondary effects (e.g. second site T-DNA insertions, mosiacism, off-target gene editing) were examined.*
